# Osteosarcoma Cell‐Derived Migrasomes Promote Macrophage M2 Polarization to Aggravate Osteosarcoma Proliferation and Metastasis

**DOI:** 10.1002/advs.202409870

**Published:** 2025-03-08

**Authors:** Wanshun Liu, Lei Li, Xiaoming Bai, Mengxue Zhang, Wei Lv, Yongbin Ma, Yuzhi Sun, Hongjing Zhang, Qing Jiang, Qingqiang Yao, Zhi‐Yuan Zhang

**Affiliations:** ^1^ Division of Sports Medicine and Adult Reconstructive Surgery Department of Orthopedic Surgery Nanjing Drum Tower Hospital Clinical College of Nanjing Medical University 321 Zhongshan Road Nanjing Jiangsu 210008 P. R. China; ^2^ School of Basic Medical Sciences Nanjing Medical University 101 Longmian Avenue Nanjing Jiangsu 211166 P. R. China; ^3^ State Key Laboratory of Pharmaceutical Biotechnology Nanjing University 22 Hankou Road Nanjing Jiangsu 210093 P. R. China; ^4^ Branch of National Clinical Research Center for Orthopedics Sports Medicine and Rehabilitation 321 Zhongshan Road Nanjing Jiangsu 210008 P. R. China; ^5^ Department of Central Laboratory Jintan Hospital Jiangsu University 500 Avenue Jintan Jintan Jiangsu 213200 P. R. China; ^6^ Department of Orthopaedic Surgery Nanjing First Hospital Nanjing Medical University 68 Changle Road Nanjing Jiangsu 210006 P. R. China

**Keywords:** macrophage, MFGE8, migrasome, osteosarcoma

## Abstract

The local tumor microenvironment (TME) of osteosarcoma (OS) includes several tumor niches that control tumor growth and cell extravasation. Migrasomes are recently discovered extracellular vesicles produced during cell migration. Herein, the results show OS cell production of migrasomes in vivo and in vitro. Osteosarcoma cell‐derived migrasomes (OCDMs) aggravate OS proliferation and metastasis, and impeding OCDM formation alleviates the malignant progression of OS. Further studies revealed that migrasome‐associated nanoparticles (MANPs) are the functional unit of OCDMs and that OCDMs promote M2 polarization of macrophages in the TME in a MANPs‐dependent manner. Moreover, milk fat globule‐EGF factor 8 (MFGE8) in OCDMs is identified as a key protein that enhances phagocytosis to promote the M2 polarization of macrophages. Overall, the results reveal that OCDMs enhance the M2 polarization of macrophages in the TME to aggravate OS progression via MFGE8. These findings may guide the development of OCDM‐modulating OS therapies.

## Graphical abstract

Migrasomes are newly discovered organelles in migrating cells. This study finds that osteosarcoma cell‐derived migrasomes promote the tumor‐promoting phenotype of macrophages and further aggravate osteosarcoma malignant progression via milk fat globule‐EGF factor 8. And migrasome‐associated nanoparticles are the key functional units for the intercellular communication role of osteosarcoma cell‐derived migrasomes.

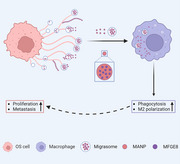

## 1 Introduction

Osteosarcoma (OS) is a malignant bone tumor originating from bone marrow mesenchymal stem cells and is prevalent in children and adolescents.^[^ ^1^ ^]^ The standard treatment for OS is surgical resection combined with chemotherapy; despite recent advances in treatments such as surgery and chemotherapy, over 30% of patients experience recurrence or metastasis, and the 5‐year survival rate for these patients is below 25%.^[^ ^2^ ^]^ Immunotherapies and targeted therapies have achieved significant efficacy in treating a wide range of tumors; however, these approaches have limited efficacy in treating OS due to the dynamic immunogenicity and genetic heterogeneity of the disease.^[^ ^3^ ^]^ Therefore, there is an urgent need to explore the underlying mechanisms of OS development and identify novel and effective therapeutic strategies.

The tumor microenvironment (TME), which includes the immune cells, peritumor vasculature, fibroblasts, various signaling molecules, and the extracellular matrix (ECM), plays an important role in tumor progression.^[^ ^4^ ^]^ Immune cells are critical components of the TME, among them monocytes and macrophages are the most predominant (>40%) immune cells in the TME,^[^ ^5^ ^]^ and moreover, the monocytes and the macrophages account for 70–80% of the total tumor‐infiltrating myeloid cells.^[^ ^6^ ^]^ Notably, macrophages in the TME exhibited a high percentage of M2 polarization, which might accelerate the malignant progression of OS. Interactions between tumor cells and immune cells in the TME supports tumor progression in this progress. On the one hand, tumor cells can recruit and reprogram monocytes and macrophages through cell‐to‐cell contact or paracrine signaling to remodel the vasculature and ECM.^[^ ^7^ ^]^ On the other hand, extracellular vesicles (EVs) released by tumor cells play an important role in orchestrating the TME and mediating interactions between tumor cells and immune cells.^[^ ^8^ ^]^ For example, metastatic OS cell‐derived exosomes orchestrate the immunosuppressive TME by enhancing the M2 polarization of tumor‐associated macrophages (TAMs), promoting OS progression.^[^ ^9^ ^]^ Therefore, targeted modulation of the TME, particularly the interaction between tumor cells and TAMs, will likely reveal new pathological mechanisms of OS progression and may lead to the discovery of new therapeutic targets.

Migrasomes are newly discovered organelles in migrating cells.^[^ ^10^ ^]^ During cell migration, migrasomes, which are vesicles of membranous structures, are formed at the tips or bifurcations of retraction fibers. A migrasome contains a variable number of smaller vesicles that constitute its characteristic morphological structure.^[^ ^10a^ ^]^ Migrasomes are connected to the cell body by retraction fibers, through which cell contents can be transported to the migrasomes and released from the cell.^[^ ^10b^ ^]^ Migrasome formation is regulated by various factors, including migration patterns, tetraspanin (TSPAN) protein, and integrin pairing with the ECM, and several studies have demonstrated that modulation of TSPAN4 expression affects migrasome formation.^[^ ^11^ ^]^ Recent studies have shown that migrasomes are important in mediating intercellular communication. During zebrafish embryonic development, migrasomes enriched with large quantities of chemokines, cytokines, and growth factors form local regional signaling centers through specific spatial and temporal distributions that regulate organ morphogenesis.^[^ ^12^ ^]^ During angiogenesis in chick embryos, monocytes act on vascular endothelial cells by releasing migrasomes enriched with angiogenic factors to promote embryonic angiogenesis.^[^ ^13^ ^]^ During tumor bone metastasis, tumor cells pass mRNA and other cytoplasmic components to osteoclasts via migrasomes, stimulating osteoclast precursor cells to differentiate into osteoclasts, which in turn secrete large quantities of acid and resorb bone to promote tumor bone metastasis.^[^ ^14^ ^]^ However, it is unclear whether migrasomes are formed in the TME of OS and whether they are involved in regulating the malignant progression of this disease.

In this study, we found that OS cells can produce migrasomes, which contribute to OS malignant progression by enhancing macrophage phagocytosis and M2 polarization. We identified migrasome‐associated nanoparticles (MANPs), revealing them to be key functional units of osteosarcoma cell‐derived migrasomes (OCDMs) that mediate communication between OS cells and macrophages. Milk fat globule‐EGF factor 8 (MFGE8) is a key protein in OCDMs and MANPs, MFGE8 knockdown in OCDMs and MANPs inhibits the tumor‐promoting effects of macrophages. Our study revealed the mechanism by which OCDMs mediate the malignant progression of OS, suggesting that targeting OCDMs to modulate the TME in OS may have substantial potential for treating this disease.

## 2 Results

### 2.1 OS Cells Produce Migrasomes

**Figure 1:**
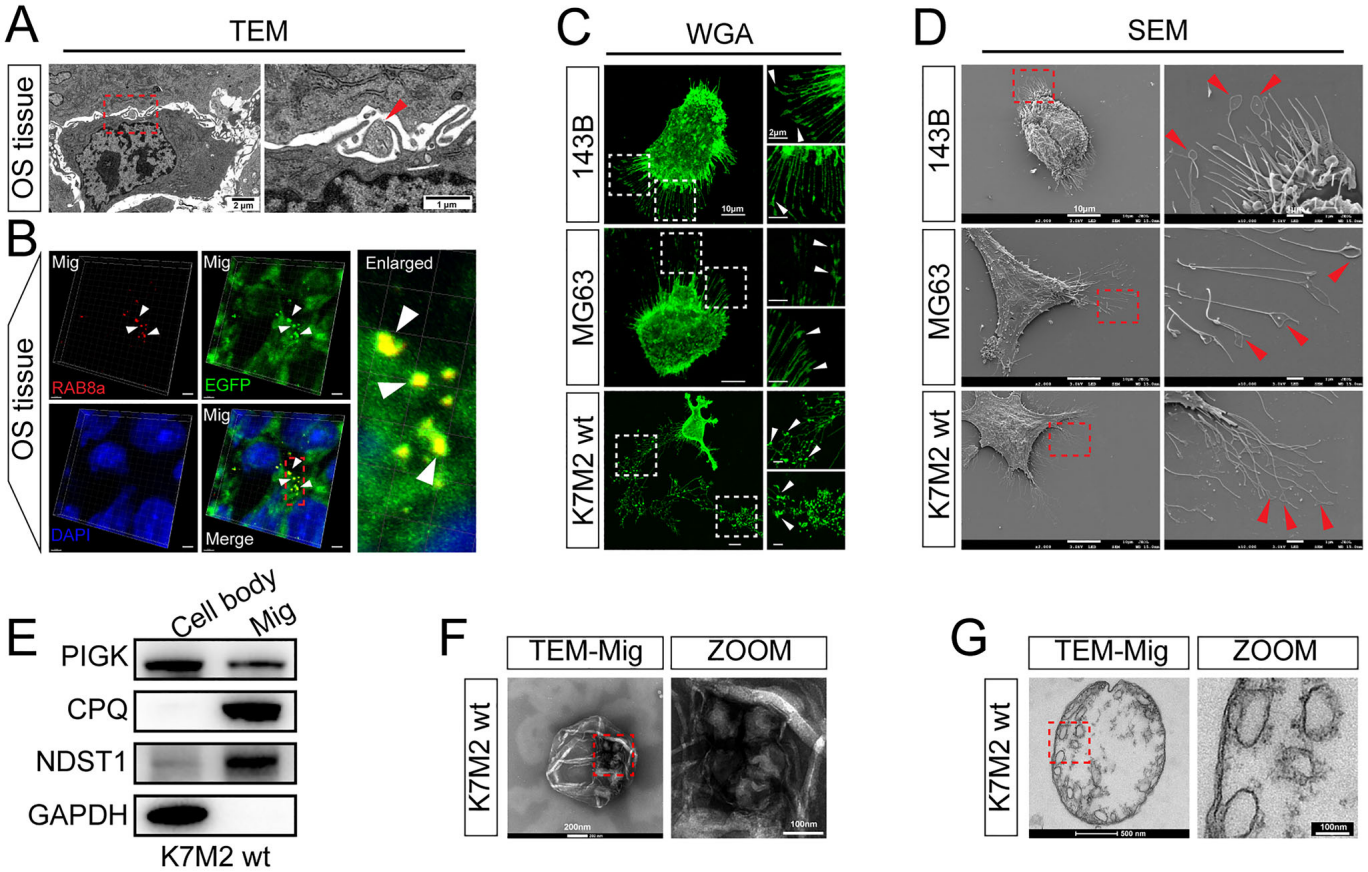
Osteosarcoma (OS) cells produce migrasomes. A) Transmission electron microscopy (TEM) image of OS tissue in BALB/c mice, with magnified image of migrasome on the right (*n* = 3). Red arrow points to migrasome. Low magnification: scale bars = 2 µm; high magnification: scale bars = 1 µm. B) Immunostaining images of RAB8a (red), TSPAN4‐EGFP^+^ OS cells (green) and DAPI (blue) in OS tissue of BALB/c mice (*n* = 3). White arrows point to migrasomes. Scale bars = 3 µm. C) Immunostaining images of Wheat‐germ agglutinin (WGA) labelled OS cells (green), with magnified images of migrasomes on the right (*n* = 3). White arrows point to the migrasomes. Low magnification: scale bars = 10 µm; high magnification: scale bars = 2 µm. D) Scanning electron microscopy (SEM) images of OS cells, with magnified images of migrasomes on the right (*n* = 3). Red arrows point to migrasomes. Low magnification: scale bars = 10 µm; high magnification: scale bars = 1 µm. E) Western blot (WB) analysis of isolated migrasomes with the indicated antibodies (*n* = 3). F) Representative TEM images of purified migrasomes by negative staining (*n* = 3). Low magnification: scale bars = 200 nm; high magnification: scale bars = 100 nm. G) Representative TEM images of purified migrasomes by ultra‐thin section (*n* = 3). Low magnification: scale bars = 500 nm; high magnification: scale bars = 100 nm.

We examined the presence of migrasomes in OS tissues using different techniques. Transmission electron microscopy (TEM) analysis of mouse OS tissues revealed the presence of typical migrasome‐like structures attached to retraction fibers in OS tissues (**Figure** **1**A). OS is highly aggressive, and migrasomes are a special class of EVs produced by cells during migration,^[^ ^10a^ ^]^ accordingly, we speculated that these migrasomes might mainly originate from OS cells in TME. Recent study reveals that RAB8a is localized on the intraluminal vesicles of migrasomes and can be used to label migrasomes.^[^ ^15^ ^]^ We next performed tumor formation experiments via using OS cells stably expressing TSPAN4‐EGFP fluorescence. Results showed that RAB8a^+^ migrasomes were predominantly colocalized with TSPAN4‐EGFP^+^ OS cells (Figure 1B). These results indicated that OS cells could produce migrasomes, and moreover, the migrasomes in TME were mainly derived from OS cells. Therefore, we further directly observed the formation of migrasomes in different OS cell lines, such as 143B, MG63, and K7M2 wt. Wheat‐germ agglutinin (WGA) is a probe for detecting migrasomes,^[^ ^16^ ^]^ we found that a large number of WGA^+^ migrasomes at the tips or bifurcations of retraction fibers in all three of the above cell lines (Figure 1C). Further scanning electron microscopy (SEM) results more clearly revealed the presence of migrasomes attached to the cell body via retraction fibers and with diameters of 0.5–3 µm at the edges of the three OS cell types (Figure 1D). In addition, we isolated migrasomes from K7M2 wt cells. Western blotting (WB) revealed the expression of migrasome markers (PIGK, CPQ, and NDST1) in the purified vesicles (Figure 1E). Moreover, the vesicles were analyzed by TEM after negative staining or ultrathin sectioning, both of which revealed that the isolated vesicles had a typical migrasome‐like structure encapsulating small vesicles, and diameter of the isolated vesicles was 0.5–3 µm (Figure 1F,G). The above results confirmed that OS cells could produce migrasomes and migrasomes in OS tissues were mainly derived from OS cells.

### 2.2 OCDMs Promote OS Proliferation and Metastasis in OS Model

**Figure 2:**
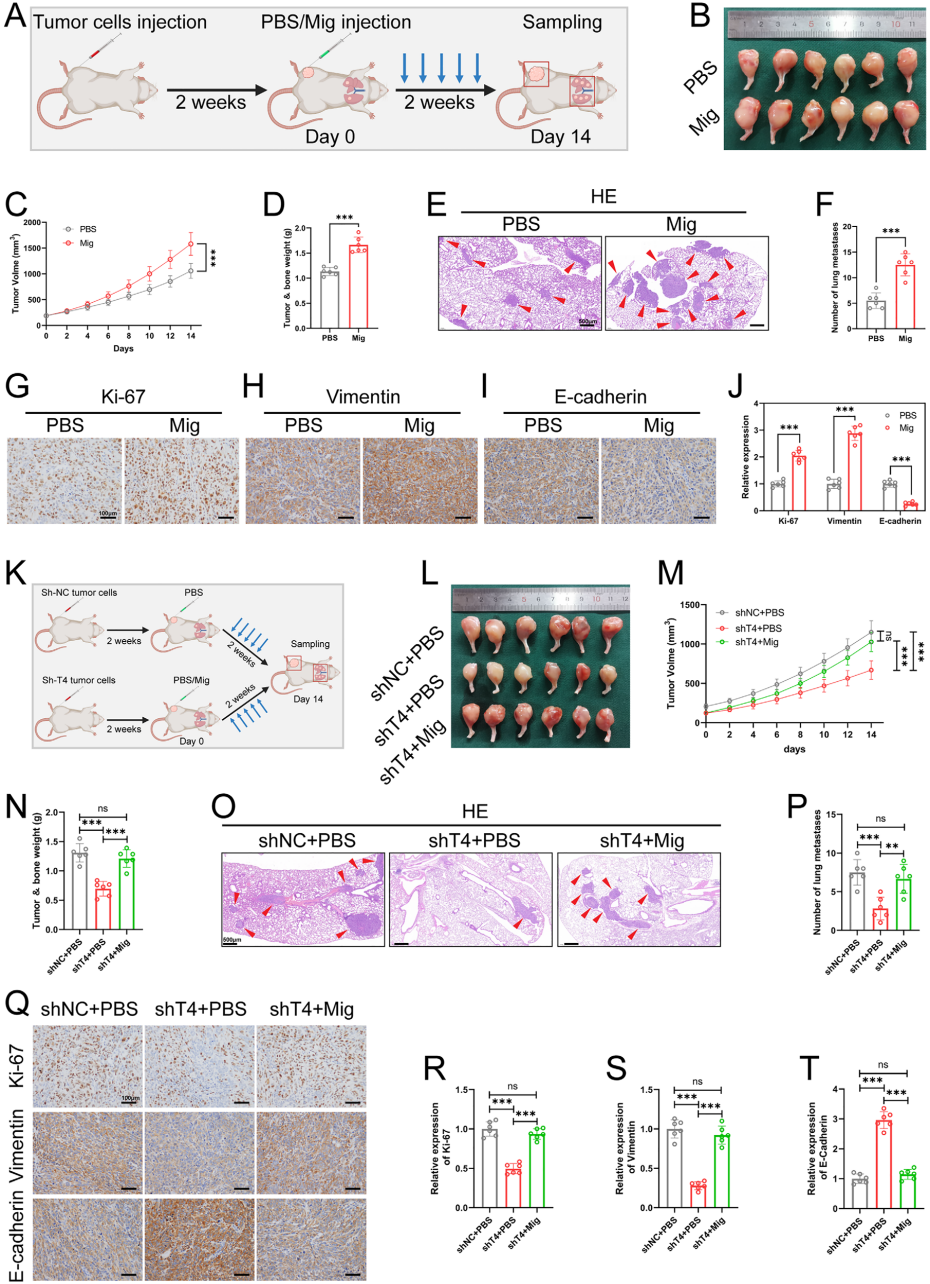
Osteosarcoma cell‐derived migrasomes (OCDMs) promote OS proliferation and metastasis in OS model. A‐J) After tibia injection of OS cells, mice were treated with PBS or migrasomes. (A) Schematic diagram of animal experiment (created with BioRender.com). (B) Representative images of tumors (*n* = 6). (C) Quantification of tumor volumes (*n* = 6). (D) Quantification of tumor and bone weight (*n* = 6). (E, F) Representative hematoxylin (HE) staining images and quantitative analysis of pulmonary metastatic nodules (*n* = 6). Red arrows point to nodules. Scale bars = 500 µm. (G‐J) Representative immunohistochemical (IHC) images and quantitative analysis of Ki67, Vimentin and E‐cadherin in tumor sections (*n* = 6). Scale bars = 100 µm. K‐T) After tibia injection of TSPAN4 knockdown or negative control OS cells, mice were treated with PBS or migrasomes. (K) Schematic diagram of animal experiment (created with BioRender.com). (L) Representative images of tumors (*n* = 6). (M) Quantification of tumor volumes (*n* = 6). (N) Quantification of tumor and bone weight (*n* = 6). (O, P) Representative HE staining images and quantitative analysis of pulmonary metastatic nodules (*n* = 6). Red arrows point to nodules. Scale bars = 500 µm. (Q‐T) Representative IHC images and quantitative analysis of Ki67, Vimentin and E‐cadherin in tumor sections (*n* = 6). Scale bars = 100 µm. Results were shown as mean ± SD. ^ns^ *p* ≥ 0.05, **p < 0.01, ***p < 0.001. One‐way ANOVA test or two‐way ANOVA test was used for multivariate analysis. Unpaired t‐tests were used for the comparison of two groups.

Given that tumor cells can regulate tumor progression through EVs, such as exosomes and apoptotic bodies, we speculated that OCDMs may be involved in and regulate the malignant progression of OS. Therefore, we established in situ tumor formation in the tibias of BALB/c mice, injected PBS or migrasomes into the tumors after 2 weeks, and assessed pathological indicators associated with OS (**Figure** **2**A). Compared with PBS injection, migrasome injection significantly increased the OS volume and weight (Figure 2B–D). Hematoxylin‐eosin (HE) staining showed that the injection of migrasomes increased the number of tumor lung metastases (Figure 2E,F). Moreover, immunohistochemical (IHC) results showed that injecting migrasomes significantly increased Ki‐67 and vimentin expression and decreased E‐cadherin expression in tumor tissues (Figure 2G–J). Our experimental results illustrate that the exogenous injection of OCDMs promotes OS proliferation and metastasis.

To further validate that OCDMs mediate OS progression, we next investigated the effect of inhibiting OCDM formation on the pathological progression of OS. We knocked down TSPAN4, a key gene that promotes the formation of migrasomes, in OS cells. The quantitative real‐time (qRT)‒PCR and WB results showed that the expression level of TSPAN4 was significantly reduced after treatment with sh‐TSPAN4 (Figure S1A–C, Supporting Information); WGA staining showed that TSPAN4 knockdown significantly reduced the number of migrasomes in OS cells (Figure S1D,E, Supporting Information). Next, after 2 weeks of in situ tumor formation in the tibia of BALB/c mice, PBS or migrasomes were injected into the tumors, and OS‐related pathological indicators were evaluated (Figure 2K). OS volume and weight were significantly lower in the sh‐TSPAN4+PBS group than those in the sh‐NC+PBS group (Figure 2L–N). HE staining revealed a significant reduction in the number of tumor lung metastases in the sh‐TSPAN4+PBS group compared to that in the sh‐NC+PBS group (Figure 2O,P). Moreover, IHC revealed significantly decreased Ki‐67 and vimentin expression and increased E‐cadherin expression in the tumor tissues of the sh‐TSPAN4+PBS group (Figure 2Q–T). Notably, migrasome treatment reversed the inhibitory effect of inhibiting OCDM formation on OS proliferation and lung metastasis (Figure 2L–T). Our results suggest that impeding OCDM formation inhibits the malignant progression of OS.

### 2.3 OCDMs Promote the M2 Polarization of Macrophages in the TME

To explore the mechanisms by which OCDMs promote OS progression, we first investigated the direct effects of OCDMs on OS cells. In coincubation experiments with OCDMs and OS cells, we observed that OS cells could take up WGA‐labeled OCDMs (Figure S2A, Supporting Information). The cell counting kit‐8 (CCK‐8) assay showed no significant difference in the OD value of OS cells after PBS or OCDM treatment (Figure S2B, Supporting Information), and the 5‐ethynyl‐2′‐deoxyuridine (EDU) assay showed no significant change in the proportion of EDU^+^ OS cells after OCDM treatment (Figure S2C,D, Supporting Information), suggesting that the OCDMs do not have a significant effect on the proliferation ability of OS cells. The Transwell assay results showed no significant difference in the number of OS cells crossing the chambers between the groups after PBS or OCDM treatment, suggesting that OCDMs do not significantly affect the migration and invasion ability of OS cells (Figure S2E–H, Supporting Information). Our experimental results indicate that OCDMs do not directly act on OS cells to promote OS proliferation and metastasis.

**Figure 3:**
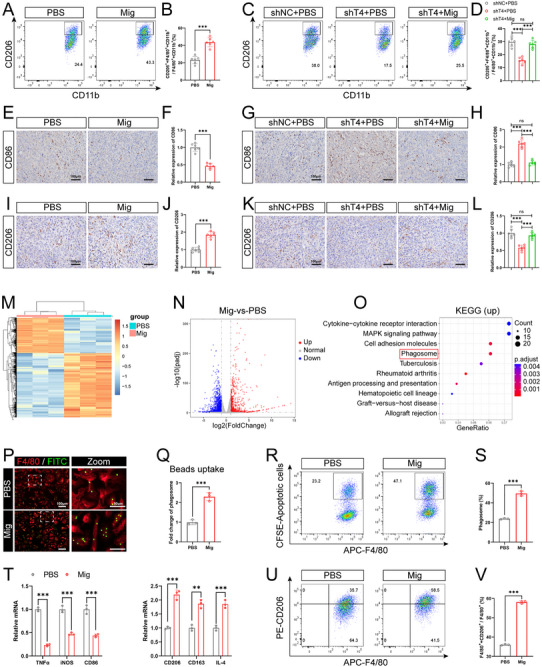
OCDMs promote macrophage phagocytosis and M2 polarization. A‐D) Flow cytometry analysis of the proportion of M2‐type macrophages in tumor tissues and quantitative analysis (*n* = 6). E‐H) Representative IHC images and quantitative analysis of CD86 in tumor sections (*n* = 6). Scale bars = 100 µm. I‐L) Representative IHC images and quantitative analysis of CD206 in tumor sections (*n* = 6). Scale bars = 100 µm. M,N) Heatmaps and volcano plots of DEGs in mRNA‑seq analysis of bone marrow‐derived macrophages (BMDMs) treated with migrasomes or PBS (*n* = 3). O) KEGG enrichment analysis of up‐regulated genes in BMDMs treated with migrasomes. P‐S) BMDMs treated with PBS or 10 µg mL^−1^ migrasomes for 24 h. (P, Q) Representative immunostaining images and quantitative analysis of BMDMs (red) phagocytosis of microbeads (green) (*n* = 3). Scale bars = 100 µm. (R, S) Flow cytometry analysis of the proportion of BMDMs (APC) that phagocytose apoptotic OS cells (CFSE) and quantitative analysis (*n* = 3). T‐V) BMDMs treated with PBS or 10 µg mL^−1^ migrasomes for 24 h, followed by co‐incubation with apoptotic OS cells for 24 h. (T) Quantitative real‐time (qRT)‐PCR analysis of the expression of M1 polarization (TNFα, iNOS and CD86) and M2 polarization markers (CD206, CD163 and IL‐4) in BMDMs (*n* = 3). (U, V) Flow cytometry analysis of the proportion of M2‐type macrophages and quantitative analysis (*n* = 3). Results were shown as mean ± SD. ^ns^ *p* ≥ 0.05, **p < 0.01, ***p < 0.001. One‐way ANOVA test or two‐way ANOVA test was used for multivariate analysis. Unpaired t‐tests were used for the comparison of two groups.

Previous studies have demonstrated that OS cells can mediate OS progression by regulating the TME,^[^ ^17^ ^]^ and we hypothesized that OCDMs may promote OS progression by regulating other cells in the TME. Therefore, we investigated the changes associated with immune cells in the TME after OCDM treatment. Flow cytometry analysis revealed no significant changes in the proportions of NK cells, neutrophils, T cells, CD8^+^ T cells, CD4^+^ T cells, Tregs, CD8/Tregs or macrophages in the OCDM‐treated OS tissues compared to those in the PBS‐treated OS tissues (Figure S3A–O, Supporting Information). Interestingly, the proportion of CD206^+^ M2‐type macrophages was significantly elevated while CD86^+^ M1‐type macrophages was significantly reduced (**Figure** **3**A,B; Figure S4A–C, Supporting Information). Correspondingly, the proportion of CD206^+^ M2‐type macrophages was significantly lower in OS tissues in the sh‐TSPAN4+PBS group than in those in the sh‐NC+PBS group, and CD86^+^ M1‐type macrophages was significantly greater in the sh‐TSPAN4+PBS group, whereas OCDM treatment rescued the proportion of M2‐type and M1‐type macrophages (Figure 3C,D; Figure S4D–F, Supporting Information). In addition, IHC revealed that the expression of CD86 was significantly lower (Figure 3E,F) and that of CD206 was significantly greater (Figure 3I,J) in the OCDM‐treated OS tissues than in the PBS‐treated OS tissues; moreover, the expression of CD86 was significantly greater (Figure 3G,H) and that of CD206 was significantly lower (Figure 3K,L) in the OS tissues in the sh‐TSPAN4+PBS group than in those in the sh‐NC+PBS group, and these effects were rescued by OCDM treatment. The above results suggest that OCDMs alter macrophage‐associated phenotypes in the TME.

Therefore, we next investigated the effects of OCDMs on macrophages. We extracted and induced mouse bone marrow‐derived macrophages (BMDMs) (Figure S5A, Supporting Information). After coincubation of OCDMs with BMDMs for 24 h, immunofluorescence detection revealed the presence of WGA‐labeled OCDMs in the BMDMs, indicating that the BMDMs had taken up the OCDMs (Figure S5B, Supporting Information). We treated BMDMs with OCDMs or PBS and performed transcriptome sequencing. There was a significant difference in RNA expression between OCDM‐treated BMDMs and PBS‐treated BMDMs. Heatmaps and volcano plots of the RNA sequencing data revealed 853 upregulated RNAs and 1609 downregulated RNAs in BMDMs after OCDMs treatment (Figure 3M,N). KEGG enrichment analyses revealed that the genes demonstrating upregulated expression in OCDM‐treated BMDMs were significantly enriched in phagosome (Figure 3O). Flow cytometry analysis revealed that the proportion of Annexin V^+^ apoptotic OS cells was significantly increased after apoptosis induction (Figure S5C,D, Supporting Information); after cytochalasin D (CyD) pretreatment of BMDMs, there was no significant difference in the proportion of CFSE^+^ macrophages that phagocytosed apoptotic cells in PBS‐BMDMs and OCDM‐BMDMs, excluding the interference of apoptotic cell adhesion (Figure S5E,F, Supporting Information). Immunostaining of FITC‐labeled microbeads coincubated with BMDMs for 1 h revealed a significant increase in the microbead/macrophage ratio after OCDM treatment (Figure 3P,Q). After coincubation of CFSE‐labeled OS cells with BMDMs for 1 h, flow cytometry analysis revealed that the proportion of CFSE^+^ macrophages that phagocytosed apoptotic cells was significantly increased in OCDM‐treated BMDMs (Figure 3R,S), suggesting that OCDMs enhance macrophage phagocytosis. Interestingly, qRT‒PCR revealed no significant changes in the expression levels of M1 polarization markers (TNFα, iNOS, and CD86) or M2 polarization markers (CD206, CD163, and IL‐4) in BMDMs after treatment with OCDMs (Figure S6A–F, Supporting Information), which appears inconsistent with the results of the in vivo experiments. Previous studies have reported that macrophages can promote M2 polarization by phagocytosing apoptotic cells; therefore, we hypothesized that OCDMs may promote M2 polarization by enhancing macrophage phagocytosis of apoptotic cells. For validation, we examined changes in the polarization ability of PBS‐BMDMs and OCDM‐BMDMs after 24 h of coculture with apoptotic OS cells. qRT‒PCR revealed that OCDM treatment downregulated the expression of M1 polarization markers (TNFα, iNOS, and CD86) and upregulated the expression of M2 polarization markers (CD206, CD163, and IL‐4) in BMDMs (Figure 3T). Flow cytometry analysis revealed that the proportion of CD206^+^ M2‐type BMDMs was significantly elevated while CD86^+^ M1‐type BMDMs was significantly reduced after OCDM treatment (Figure 3U,V; Figure S7A–C, Supporting Information). In addition, qRT‒PCR showed no significant difference in the polarization capacity of PBS‐BMDMs and OCDM‐BMDMs cocultured with apoptotic OS cells for 24 h after CyD pretreatment of BMDMs (Figure S6G–L, Supporting Information). Our experimental results illustrate that OCDMs enhance macrophage phagocytosis and further promote macrophage M2 polarization through this mechanism. Previous studies have reported that CXCL9:SPP1 macrophage polarity is a good choice for selecting anti‐ or pro‐tumor macrophages in sarcoma,^[^ ^18^ ^]^ we also examined changes in the CXCL9:SPP1 polarity of PBS‐BMDMs and OCDM‐BMDMs after 24 h of coculture with apoptotic OS cells. qRT‒PCR revealed that OCDM treatment downregulated the expression of CXCL9 and upregulated the expression of SPP1 in BMDMs (Figure S7D–F, Supporting Information). Our results suggest that OCDMs inhibit CXCL9:SPP1 macrophage polarity and promote the transformation of macrophages into pro‐tumor macrophages.

To further investigate whether OCDMs promote OS progression by regulating macrophages, we investigated the phenotypic changes in OS cells after coculture with macrophages using a coculture chamber model (Figure S8A, Supporting Information). The results of the CCK‐8 and EDU experiments showed that the proliferation ability of OS cells was significantly greater after coculture with OCDM‐BMDMs than after coculture with PBS‐ BMDMs (Figure S8B–D, Supporting Information), and the Transwell results showed that the migration and invasion abilities of OS cells were significantly greater after coculture with OCDM‐BMDMs (Figure S8E–H, Supporting Information), consistent with the in vivo findings.

### 2.4 OCDMs Promote Macrophage Phagocytosis and M2 Polarization via MANPs

**Figure 4:**
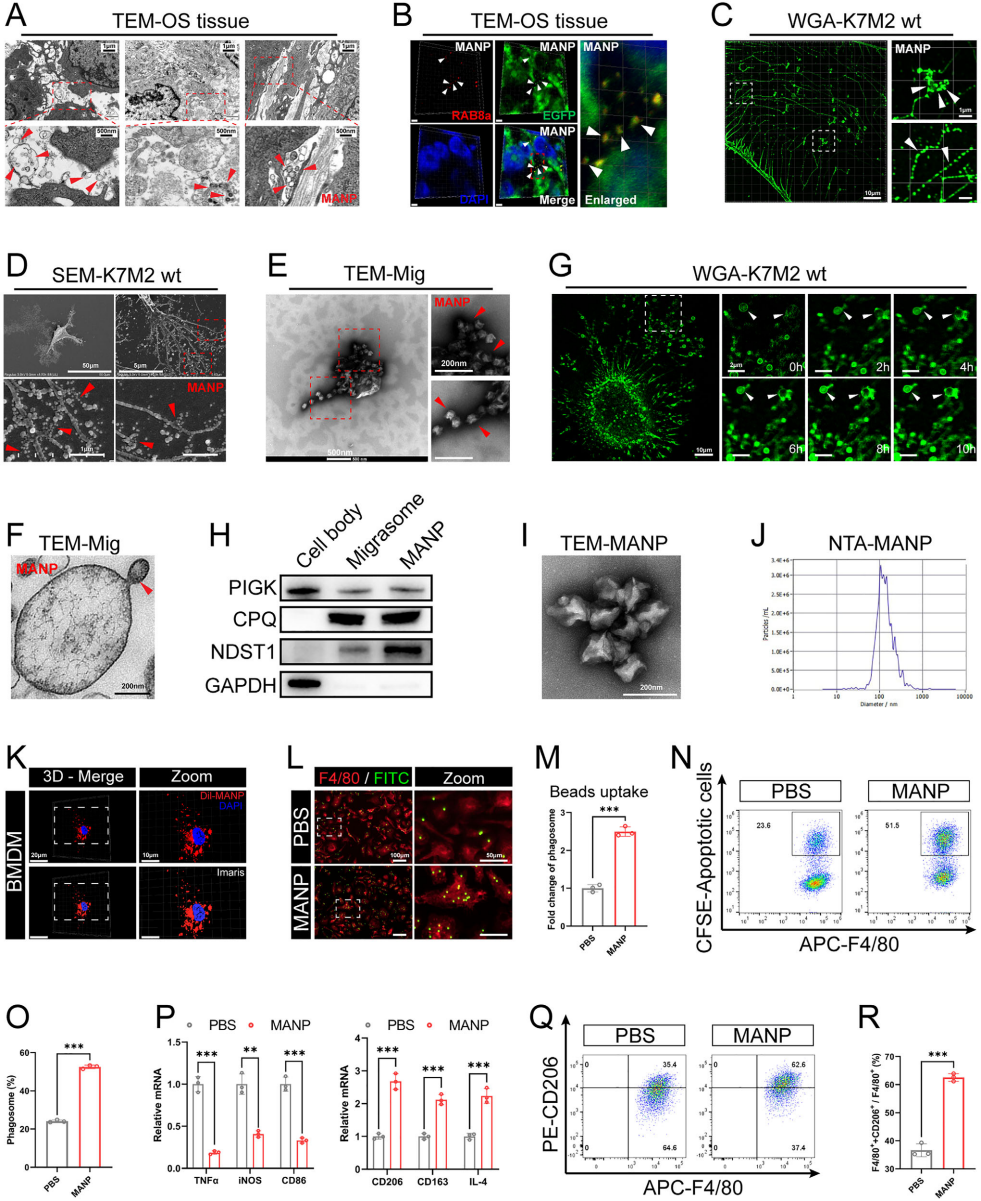
Migrasome‐associated nanoparticles (MANPs) promote macrophage phagocytosis and M2 polarization. A) TEM image of OS tissue in BALB/c mice (*n* = 3). Red arrow points to MANPs. Low magnification: scale bars = 4 µm; high magnification: scale bars = 250 nm. B) Immunostaining images of RAB8a (red), TSPAN4‐EGFP^+^ OS cells (green) and DAPI (blue) in OS tissue of BALB/c mice (*n* = 3). White arrows point to MANPs. Scale bars = 3 µm. C) Immunostaining images of WGA labelled OS cells (*n* = 3). Low magnification: scale bars = 10 µm; high magnification: scale bars = 1 µm. D) SEM images of OS cells (*n* = 3). Low magnification: scale bars = 50 µm; high magnification: scale bars = 1 µm. E) Representative TEM images of purified migrasome by negative staining (*n* = 3). Low magnification: scale bars = 500 nm; high magnification: scale bars = 200 nm. F) Representative TEM images of purified migrasomes by ultra‐thin section (*n* = 3). G) Confocal time series images of WGA (green) labelled OS cells (*n* = 3). White arrows point to the migrasome releasing the MANPs. Low magnification: scale bars = 10 µm; high magnification: scale bars = 2 µm. H) WB analysis of isolated MANPs with the indicated antibodies (*n* = 3). I) Representative TEM images of purified MANPs by negative staining (*n* = 3). J) Nanoparticle tracking analysis (NTA) of purified MANPs (*n* = 3). K) Representative immunostaining images of BMDMs phagocytosis of MANPs (red) (*n* = 3). Low magnification: scale bars = 20 µm; high magnification: scale bars = 10 µm. L‐O) BMDMs treated with PBS or 10 µg mL^−1^ MANPs for 24 h. (L, M) Representative immunostaining images and quantitative analysis of BMDMs phagocytosis of microbeads (*n* = 3). (N, O) Flow cytometry analysis of the proportion of BMDMs that phagocytose apoptotic OS cells and quantitative analysis (*n* = 3). P‐R) BMDMs treated with PBS or 10 µg mL^−1^ MANPs for 24 h, followed by co‐incubation with apoptotic OS cells for 24 h. (P) qRT‐PCR analysis of the expression of M1 and M2 polarization markers in BMDMs (*n* = 3). (Q, R) Flow cytometry analysis of the proportion of M2‐type macrophages and quantitative analysis (*n* = 3). Results were shown as mean ± SD. **p < 0.01, ***p < 0.001. Unpaired t‐tests were used for the comparison of two groups.

Previous studies have revealed the presence of a variable number of small vesicles with diameters ranging from 50 to 250 nm within the migrasome, and our previous study suggested that such vesicles may be the functional unit of migrasome function.^[^ ^19^ ^]^ TEM analysis of mouse OS tissue revealed the presence of migrasomes, small vesicles released by migrasomes and small vesicles located within retraction fibers (**Figure** **4**A). Tissue immunostaining revealed that RAB8a^+^ small vesicles were predominantly colocalized with TSPAN4‐EGFP^+^ OS cells (Figure 4B). These results indicated that OS cells could produce small vesicles, and moreover, the small vesicles in TME were mainly derived from OS cells. WGA staining of OS cells revealed that either migrasome rupture or retraction fiber breakage released WGA^+^ small vesicles (Figure 4C). Moreover, SEM analyses revealed that the rupture of migrasome or retraction fibers of OS cells could produce a large number of small vesicles with diameters of 50–250 nm (Figure 4D). Notably, TEM of the purified migrasomes after negative staining revealed that the morphology and size of the small vesicles released by migrasome rupture and retraction fiber breakage were identical, suggesting that they were the same type of small vesicles (Figure 4E). TEM of the ultrathin sections of the purified migrasomes revealed that the migrasomes also released small vesicles by budding (Figure 4F). Moreover, live‐cell imaging analysis of OS cells after WGA staining revealed the release of small vesicles from the migrasomes through budding (Figure 4G). Our data suggest that small vesicles can be produced in three ways—migrasome rupture, retraction fiber breakdown, and migrasome budding—and considering the correlation between small vesicles and migrasomes, we named the small vesicles “MANPs”. Previous studies have demonstrated that migrasomes play an important role in intercellular information transfer; however, the average life cycle of migrasomes is only ≈400 min, and the membrane structure of migrasomes is prone to rupture after maturation or during purification.^[^ ^20^ ^]^ Accordingly, we speculate that MANP, which has a membrane structure, may be the functional unit of the information transfer function of the migrasome.

Therefore, we next investigated whether MANP is a key functional unit of OCDM in regulating the macrophage‐mediated malignant progression of OS. We first purified MANPs from K7M2 wt cells. WB confirmed that migrasome markers (PIGK, CPQ, and NDST1) were also expressed in purified MANPs (Figure 4H). MANPs were negatively stained and analyzed by TEM, revealing that although the sizes of the MANPs were similar to those of the exosomes, they were morphologically distinct from those of the exosomes with cup‐like structures, and the surfaces of the MANPs were wrinkled and morphologically irregular (Figure 4I). Nanoparticle tracking analysis (NTA) showed that the average particle size of the MANPs was 150.9 nm, consistent with the 50–250 nm diameter reported in previous studies (Figure 4J). We then investigated the effect of MANPs on the phagocytosis and polarization of BMDMs. After the coincubation of MANPs with BMDMs for 24 h, immunofluorescence detection revealed the presence of Dil‐labeled MANPs in BMDMs, suggesting the uptake of MANPs by BMDMs (Figure 4K). After coincubation with FITC‐labeled microbeads and BMDMs for 1 h, immunostaining revealed that the microbead/macrophage ratio was significantly increased after MANP treatment (Figure 4L,M). After coincubation with CFSE‐labeled apoptotic OS cells and BMDMs for 1 h, flow cytometric analysis revealed that the proportion of CFSE^+^ macrophages that phagocytosed apoptotic OS cells was significantly increased in the MANP‐treated BMDMs (Figure 4N,O), suggesting that the phagocytic ability of macrophages was enhanced by MANPs. After 24 h of coculture with apoptotic OS cells, qRT‒PCR revealed that MANP treatment downregulated the expression of M1 polarization markers (TNFα, iNOS, and CD86) and CXCL9, while upregulated the expression of M2 polarization markers (CD206, CD163, and IL‐4) and SPP1 in BMDMs (Figure 4P; Figure S9A–C, Supporting Information). Flow cytometric analysis revealed that the proportion of CD206^+^ M2‐type BMDMs was significantly elevated while CD86^+^ M1‐type BMDMs was significantly reduced after MANP treatment (Figure 4Q,R; Figure S9D–F, Supporting Information), demonstrating that MANPs promote macrophage M2 polarization and inhibit CXCL9:SPP1 macrophage polarity. Our results suggest that, similar to the effects of OCDMs on macrophages, MANPs also enhance macrophage phagocytosis, promote macrophage M2 polarization and facilitate the conversion of macrophage to pro‐tumor macrophage.

**Figure 5:**
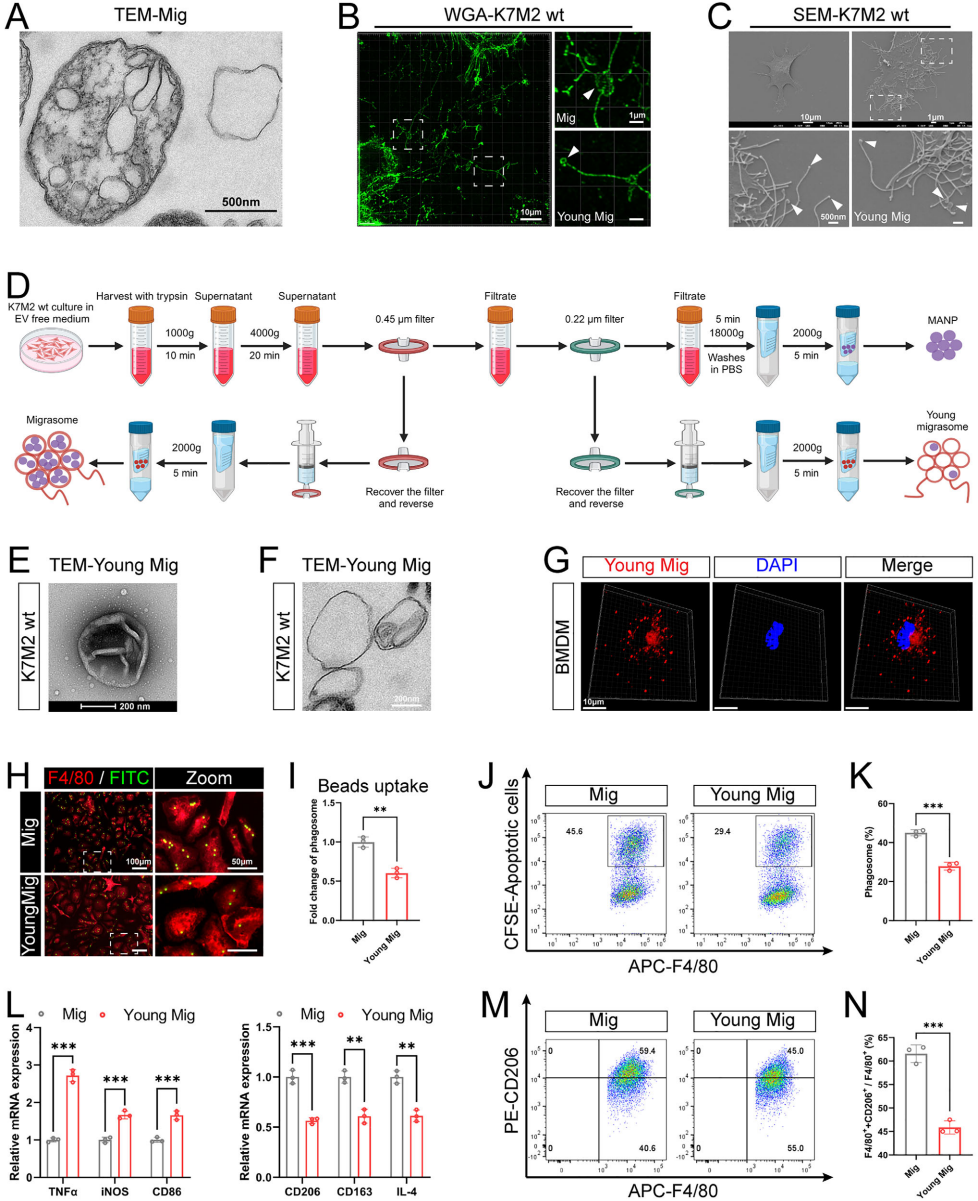
OCDMs promote macrophage phagocytosis and M2 polarization via MANPs. A) Representative TEM images of purified migrasomes by ultra‐thin section (*n* = 3). Scale bars = 500 nm. B) Immunostaining images of WGA labelled OS cells, with magnified images of migrasome and young migrasome on the right (*n* = 3). White arrows point to the migrasome and the young migrasome. Low magnification: scale bars = 10 µm; high magnification: scale bars = 1 µm. C) SEM images of OS cells (*n* = 3). Red arrows point to young migrasomes. Low magnification: scale bars = 10 µm; high magnification: scale bars = 500 nm. D) Schematic diagram of migrasomes, MANPs and young migrasomes purification (created with BioRender.com). E) Representative TEM images of purified young migrasomes by negative staining (*n* = 3). Scale bars = 200 nm. F) Representative TEM images of purified young migrasomes by ultra‐thin section (*n* = 3). Scale bars = 200 nm. G) Representative immunostaining images of BMDMs phagocytosis of young migrasomes (red) (*n* = 3). Scale bars = 10 µm. H‐K) BMDMs treated with 10 µg mL^−1^ migrasomes or young migrasomes for 24 h. (H, I) Representative immunostaining images and quantitative analysis of BMDMs (red) phagocytosis of microbeads (green) (*n* = 3). Low magnification: scale bars = 100 µm; high magnification: scale bars = 50 µm. (J, K) Flow cytometry analysis of the proportion of BMDMs (APC) that phagocytose apoptotic OS cells (CFSE) and quantitative analysis (*n* = 3). L‐N) BMDMs treated with 10 µg mL^−1^ migrasomes or young migrasomes for 24 h, followed by co‐incubation with apoptotic OS cells for 24 h. (L) qRT‐PCR analysis of the expression of M1 polarization and M2 polarization markers in BMDMs (*n* = 3). (M, N) Flow cytometry analysis of the proportion of M2‐type macrophages and quantitative analysis (*n* = 3). Results were shown as mean ± SD. **p < 0.01, ***p < 0.001. Unpaired t‐tests were used for the comparison of two groups.

Next, we investigated whether OCDMs regulate macrophage phagocytosis and polarization through MANPs. Recent studies have revealed that the number of small vesicles in the migrasome is positively correlated with the migrasome diameter, and migrasome formation involves two stages: the young migrasome and the mature migrasome, in which the young migrasome has a smaller diameter.^[^ ^11^, ^13^ ^]^ We observed the purified migrasomes by TEM and found that the number of small vesicles in the ∼0.5 µm diameter migrasomes was lower than those of typical 0.5–3 µm migrasomes (**Figure** **5**A). This finding suggested that MANPs were lacking in young migrasomes. After WGA staining of OS cells, we detected a large number of young migrasomes with diameters under 0.5 µm in addition to the typical migrasomes with diameters of 0.5–3 µm (Figure 5B). Similarly, SEM analyses revealed that OS cells can produce a large number of young migrasomes with diameters under 0.5 µm (Figure 5C). We constructed a purification system for young migrasomes and isolated them (Figure 5D). TEM analysis of young migrasomes after negative staining revealed that they were morphologically similar to migrasomes in that they were round vesicles with wrinkled membrane surfaces, but their diameters were smaller than those of typical 0.5–3 µm migrasomes (Figure 5E). TEM analysis of ultrathin sections revealed a lack of small vesicles inside young migrasomes (Figure 5F; Figure S10, Supporting Information). The above results suggest a lack of MANPs in young migrasomes; therefore, we used young migrasomes for further rescue experiments.

After coincubation of BMDMs with young migrasomes for 24 h, immunofluorescence revealed the presence of DiI‐labeled young migrasomes in the BMDMs, indicating that the BMDMs had taken up the young migrasomes (Figure 5G). After coincubation of the BMDMs with FITC‐labeled microbeads for 1 h, immunostaining revealed that the microbead/macrophage ratio was significantly lower after young migrasome treatment than after migrasome treatment (Figure 5H,I). After coincubation of the CFSE‐labeled apoptotic OS cells with the BMDMs for 1 h, flow cytometry analysis revealed a significant decrease in the proportion of CFSE^+^ macrophages that phagocytosed apoptotic OS cells among the young migrasome‐treated BMDMs compared with that in the migrasome‐treated BMDMs (Figure 5J,K). After 24 h of coculture with apoptotic OS cells, qRT‒PCR revealed upregulation of the expression of M1 polarization markers (TNFα, iNOS, and CD86) and CXCL9, while downregulation of the expression of M2 polarization markers (CD206, CD163, and IL‐4) and SPP1 in BMDMs after young migrasome treatment compared to that in those after migrasome treatment (Figure 5L; Figure S11A–C, Supporting Information). Flow cytometry analysis revealed a significant reduction in the proportion of CD206^+^ M2‐type BMDMs and significant elevation in the proportion of CD86^+^ M1‐type BMDMs after young migrasome treatment compared to that after migrasome treatment (Figure 5M,N; Figure S11D–F, Supporting Information). These results indicated that phagocytosis and M2 polarization were significantly reduced while CXCL9:SPP1 polarity was significantly elevated in BMDMs treated with young migrasomes lacking MANPs compared to those observed in BMDMs treated with migrasomes. This finding suggests that OCDMs regulate macrophage phagocytosis and polarization through MANPs.

### 2.5 OCDMs and MANPs Promote Macrophage Phagocytosis and M2 Polarization via MFGE8

**Figure 6:**
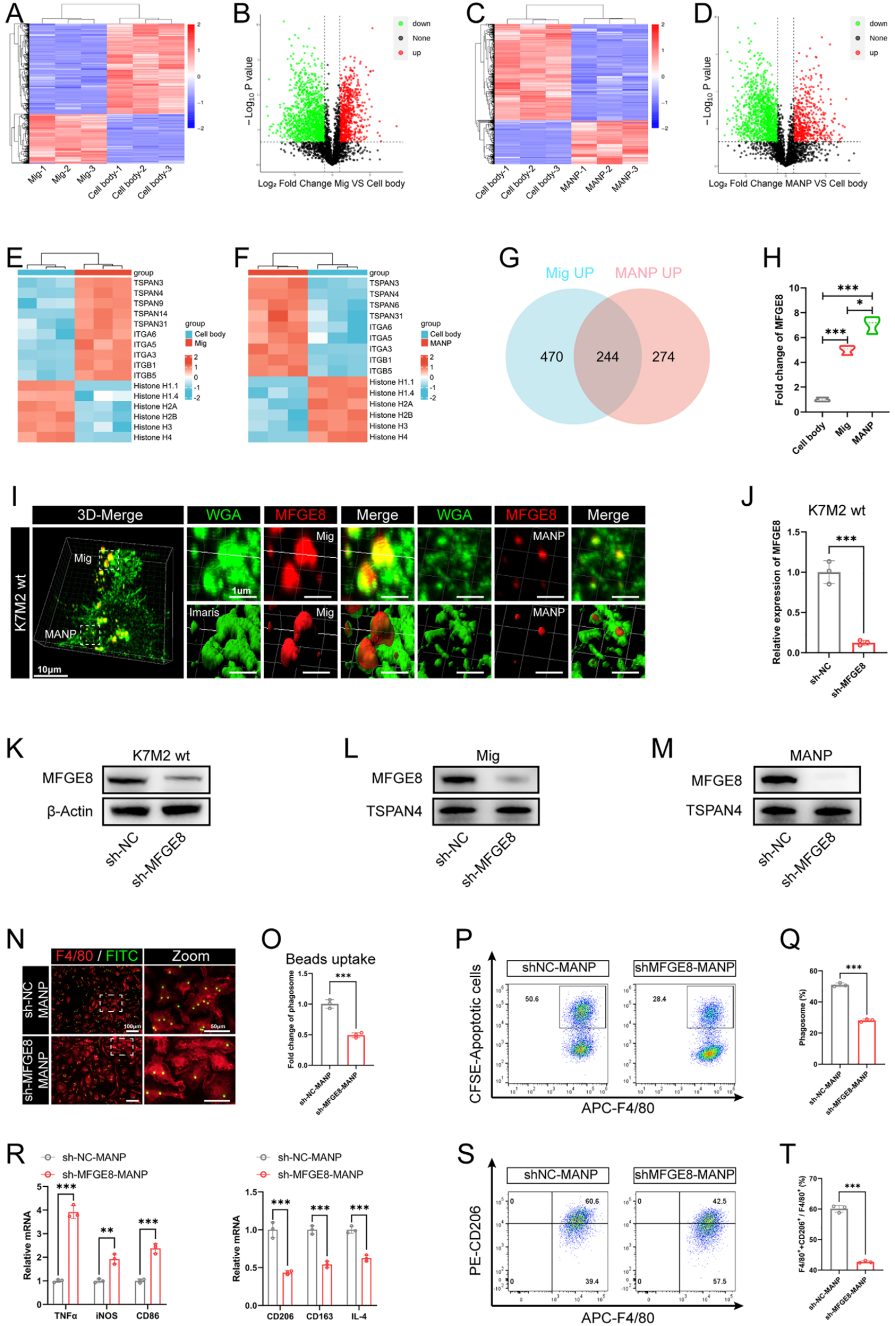
MANPs promote macrophage phagocytosis and M2 polarization via Milk fat globule‐EGF factor 8 (MFGE8). A‐H) 4D proteome sequencing analysis of cell body, migrasome and MANP. (A‐D) Heatmaps and volcano plots of DEGs in protein‑seq analysis of cell body, migrasome and MANP (*n* = 3). (E, F) Heatmaps of TSPAN proteins, integrin proteins, and histones in cell body, migrasome and MANP (*n* = 3). (G) Venn diagram showing proteins co‐enriched by migrasomes and MANPs. (H) Relative expression of MFGE8 in cell body, migrasome and MANP (*n* = 3). I) Immunostaining images of WGA (green) and MFGE8 (red) in OS cells (*n* = 3). Magnified images of migrasomes and MANPs are shown on the right. Low magnification: scale bars = 10 µm; high magnification: scale bars = 1 µm. J,K) qRT‐PCR and WB analysis of MFGE8 knockdown efficiency in K7M2 wt cells (*n* = 3). L,M) WB analysis of MFGE8 knockdown efficiency in migrasomes and MANPs (*n* = 3). N‐Q) BMDMs treated with 10 µg mL^−1^ sh‐NC MANPs or sh‐MFGE8 MANPs for 24 h. (N, O) Representative immunostaining images and quantitative analysis of BMDMs (red) phagocytosis of microbeads (green) (*n* = 3). Low magnification: scale bars = 100 µm; high magnification: scale bars = 50 µm. (P, Q) Flow cytometry analysis of the proportion of BMDMs (APC) that phagocytose apoptotic OS cells (CFSE) and quantitative analysis (*n* = 3). R‐T) BMDMs treated with 10 µg mL^−1^ sh‐NC MANPs or sh‐MFGE8 MANPs for 24 h, followed by co‐incubation with apoptotic OS cells for 24 h. (R) qRT‐PCR analysis of the expression of M1 polarization and M2 polarization markers in BMDMs (*n* = 3). (S, T) Flow cytometry analysis of the proportion of M2‐type macrophages and quantitative analysis (*n* = 3). Results were shown as mean ± SD. *p < 0.05, **p < 0.01, ***p < 0.001. One‐way ANOVA test or two‐way ANOVA test was used for multivariate analysis. Unpaired t‐tests were used for the comparison of two groups.

To understand the molecular mechanism of OCDM and MANP regulation in macrophages, we performed 4D proteome sequencing of the OS cell body, migrasomes, and MANPs. The heatmaps and volcano maps generated from the sequencing data revealed 714 upregulated and 1870 downregulated proteins in the migrasomes (**Figure** **6**A,B) and 518 upregulated and 2056 downregulated proteins in the MANPs compared to those in the cell body (Figure 6C,D). Heatmaps showed that known migrasome‐enriched proteins, such as TSPANs and integrin β, were enriched in migrasomes and MANPs, whereas nuclear proteins were significantly reduced (Figure 6E,F), suggesting that 4D proteome sequencing analyses were reliable. To screen for key proteins regulating macrophages in OCDMs and MANPs, we performed an intersection analysis of upregulated proteins in the migrasomes and MANPs, and a Venn diagram showed that 244 proteins were significantly upregulated in both the migrasomes and MANPs (Figure 6G). Notably, MFGE8 was among the 244 upregulated proteins, and its expression level was greater in MANPs than in migrasomes (Figure 6H). Previous studies have shown that MFGE8, a secreted glycoprotein that enhances macrophage phagocytosis of apoptotic cells and promotes macrophage M2 polarization, plays an important role in macrophage biology.^[^ ^21^ ^]^ Therefore, we hypothesized that OCDMs and MANPs may regulate macrophages by enriching MFGE8.

Next, we investigated whether OCDMs and MANPs regulate macrophage phagocytosis and polarization function by enriching MFGE8. Immunofluorescence staining of OS cells for MFGE8 and WGA revealed that the MFGE8 protein colocalized with WGA^+^ migrasomes and MANPs, suggesting that the MFGE8 protein is enriched in OCDMs and MANPs (Figure 6I). Subsequently, we knocked down MFGE8 in OS cells with short hairpin RNA (shRNA) and screened OS cells with stable MFGE8 knockdown with Puromycin. The qRT‒PCR and WB results showed that the expression level of MFGE8 was significantly reduced in OS cells after MFGE8 knockdown (Figure 6J,K). Moreover, WB revealed that the expression level of MFGE8 was significantly lower in the migrasomes and MANPs of OS cell lines with stable knockdown of MFGE8 than in the migrasomes and MANPs of control cell lines (Figure 6L,M). The above results indicated that we successfully knocked down MFGE8 in migrasomes and MANPs. Next, we performed rescue experiments using sh‐MFGE8‐MANPs. After coincubation of FITC‐labeled microbeads with BMDMs for 1 h, immunostaining revealed that the microbead/macrophage ratio was significantly lower after sh‐MFGE8‐MANP treatment than after sh‐NC‐MANP treatment (Figure 6N,O). After coincubation of CFSE‐labeled apoptotic OS cells with BMDMs for 1 h, flow cytometry analysis revealed a significant decrease in the proportion of CFSE^+^ macrophages that phagocytosed apoptotic OS cells in sh‐MFGE8‐MANP‐treated BMDMs compared with that in sh‐NC‐MANP‐treated BMDMs (Figure 6P,Q). After 24 h of coculture with apoptotic OS cells, qRT‒PCR revealed upregulation of the expression of M1 polarization markers (TNFα, iNOS, and CD86) and CXCL9, while downregulation of the expression of M2 polarization markers (CD206, CD163, and IL‐4) and SPP1 in BMDMs after sh‐MFGE8‐MANP treatment compared to those after sh‐NC‐MANP treatment (Figure 6R; Figure S12A–C, Supporting Information); flow cytometry analysis revealed a significant reduction in the proportion of CD206^+^ M2‐type BMDMs and significant elevation in the proportion of CD86^+^ M1‐type BMDMs after sh‐MFGE8‐MANP treatment compared to that after sh‐NC‐MANP treatment (Figure 6S,T; Figure S12D–F, Supporting Information). The above results indicated that phagocytosis and M2 polarization were significantly lower while CXCL9:SPP1 polarity was significantly higher in BMDMs treated with sh‐MFGE8‐MANPs than in those treated with sh‐NC‐MANPs. This finding suggests that MANPs regulate macrophage phagocytosis and polarization through MFGE8.

**Figure 7:**
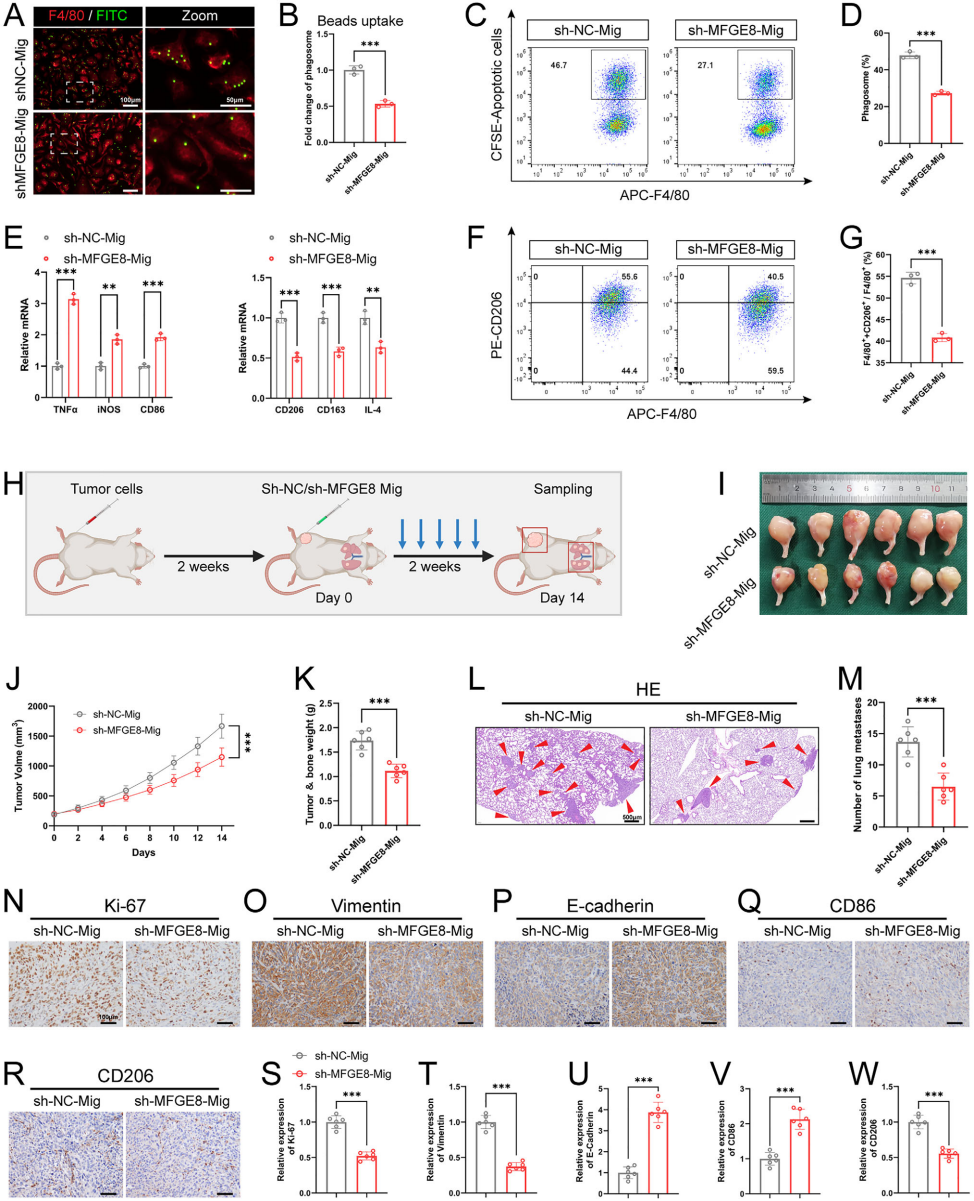
OCDMs promote OS progression through MFGE8. A‐D) BMDMs treated with 10 µg mL^−1^ sh‐NC migrasomes or sh‐MFGE8 migrasomes for 24 h. (A, B) Representative immunostaining images and quantitative analysis of BMDMs (red) phagocytosis of microbeads (green) (*n* = 3). Low magnification: scale bars = 100 µm; high magnification: scale bars = 50 µm. (C, D) Flow cytometry analysis of the proportion of BMDMs (APC) that phagocytose apoptotic OS cells (CFSE) and quantitative analysis (*n* = 3). E‐G) BMDMs treated with 10 µg mL^−1^ sh‐NC migrasomes or sh‐MFGE8 migrasomes for 24 h, followed by co‐incubation with apoptotic OS cells for 24 h. (E) qRT‐PCR analysis of the expression of M1 polarization and M2 polarization markers in BMDMs (*n* = 3). (F, G) Flow cytometry analysis of the proportion of M2‐type macrophages and quantitative analysis (*n* = 3). H‐W) After tibia injection of OS cells, mice were treated with sh‐NC migrasomes or sh‐MFGE8 migrasomes. (H) Schematic diagram of animal experiment (created with BioRender.com). (I) Representative images of tumors (*n* = 6). (J) Quantification of tumor volumes (*n* = 6). (K) Quantification of tumor and bone weight (*n* = 6). (L, M) Representative HE staining images and quantitative analysis of pulmonary metastatic nodules (*n* = 6). Scale bars = 500 µm. (N‐W) Representative IHC images and quantitative analysis of Ki67, Vimentin and E‐cadherin in tumor sections (*n* = 6). Scale bars = 100 µm. Results were shown as mean ± SD. **p < 0.01, ***p < 0.001. One‐way ANOVA test or two‐way ANOVA test was used for multivariate analysis. Unpaired t‐tests were used for the comparison of two groups.

To investigate whether OCDMs also regulate macrophage phagocytosis and polarization through MFGE8, we performed rescue experiments using sh‐MFGE8 migrasomes. After coincubation of FITC‐labeled microbeads with BMDMs for 1 h, immunostaining revealed that the microbead/macrophage ratio was significantly lower after sh‐MFGE8 migrasome treatment than after sh‐NC migrasome treatment (**Figure** **7**A,B). After coincubation of CFSE‐labeled apoptotic OS cells with BMDMs for 1 h, flow cytometry analysis revealed a significant decrease in the proportion of CFSE^+^ macrophages that phagocytosed apoptotic OS cells in sh‐MFGE8 migrasome‐treated BMDMs compared with that in sh‐NC migrasome‐treated BMDMs (Figure 7C,D). After 24 h of coculture with apoptotic OS cells, qRT‒PCR revealed upregulation of the expression of M1 polarization markers (TNFα, iNOS, and CD86) and CXCL9, while downregulation of the expression of M2 polarization markers (CD206, CD163, and IL‐4) and SPP1 in BMDMs after sh‐MFGE8 migrasome treatment compared to those after sh‐NC migrasome treatment (Figure 7E; Figure S13A–C, Supporting Information). Flow cytometry analysis revealed a significant reduction in the proportion of CD206^+^ M2‐type BMDMs and significant elevation in the proportion of CD86^+^ M1‐type BMDMs after sh‐MFGE8 migrasome treatment compared to that after sh‐NC migrasome treatment (Figure 7F,G; Figure S13D–F, Supporting Information). The above results indicated that phagocytosis and M2 polarization were significantly lower while CXCL9:SPP1 polarity was significantly higher in BMDMs treated with sh‐MFGE8 migrasomes than in those treated with sh‐NC migrasomes. This finding suggests that OCDMs regulate macrophage phagocytosis and polarization through MFGE8.

We found that OCDMs promote OS progression by enhancing macrophage phagocytosis and promoting M2 polarization and that the regulatory effects of OCDMs on macrophage phagocytosis and polarization depend on MFGE8. Therefore, we hypothesized that MFGE8 is involved in OCDM promotion of OS progression. To validate the role of MFGE8 in OCDMs in promoting OS progression, we next investigated the effect of the knockdown of MFGE8 in migrasomes on OS pathological progression. After 2 weeks of in situ tumor formation in the tibia of BALB/c mice, sh‐MFGE8 migrasomes or sh‐NC migrasomes were injected into the tumors, and OS‐related pathological indicators were evaluated (Figure 7H). OS volume and weight were significantly lower in the sh‐MFGE8 migrasome group than in the sh‐NC migrasome group (Figure 7I–K). HE staining revealed a significant reduction in the number of tumor lung metastases in the sh‐MFGE8‐treated group compared to that in the sh‐NC‐treated group (Figure 7L,M). Moreover, the IHC results showed that the expression levels of Ki‐67, vimentin and CD206 were significantly lower and that the expression levels of E‐cadherin and CD86 were significantly greater in the tumor tissues of the sh‐MFGE8 migrasome group than in those of the sh‐NC migrasome group (Figure 7N–W). The above results indicate that MFGE8 knockdown in OCDMs inhibits the promotion of OS progression by OCDMs. This finding suggests that OCDMs promote OS malignant progression through MFGE8.

## 3 Discussion

The TME is closely related to tumor progression, and tumor cells can affect their microenvironment by releasing extracellular vesicles, promoting tumor proliferation and metastasis.^[^ ^9^ ^]^ Migrasomes regulate the biological functions of recipient cells by transporting their contents to them and play an important role in cell communication in physiological and pathological processes.^[^ ^12^ ^]^ This study demonstrated that OCDMs and MANPs enhance macrophage phagocytosis and promote its further M2 polarization by enriching MFGE8, thereby promoting OS proliferation and metastasis.

Migrasomes are single‐membrane vesicles with diameters of 0.5–3.0 µm formed at the end or bifurcation of retraction fibers of cells during migration; these vesicles are widely found in all types of migrating cells and are rich in a varying number of small vesicles.^[^ ^10b^ ^]^ The pairing of integrins with matched ECM ligand, enriching of TSPAN4 and cell migration patterns regulate migrasome formation (Figure S14A–C, Supporting Information).^[^ ^11a,b,d^ ^]^ In addition, our results show that SAR407899 can inhibit the formation of OCDMs (Figure S15A–F, Supporting Information). Cells continuously transport intracellular material into migrasomes during migration through retraction fibers, which subsequently break, releasing migrasomes that play an important role in intercellular communication by being taken up and utilized by other cells.^[^ ^20^ ^]^ Tumor cells are likely an important source of migrasomes due to their strong migratory ability. Previous studies have demonstrated that tumor cells pass mRNA and other cytoplasmic components to osteoclasts via migrasomes, stimulating osteoclast precursor cells to differentiate into osteoclasts in an abnormal state, which then secrete large quantities of acid and resorb bone to promote bone metastasis of tumors.^[^ ^14^ ^]^ In this study, our results multi‐dimensionally demonstrate that OS cells could produce migrasomes. In mouse and human OS cell lines, a large number of migrasomes with diameters of 0.5–3.0 µm at the ends of the retraction fibers. In vivo, our TEM images clearly showed the migrasomes were existed in TME, and importantly, most of RAB8a^+^ migrasomes were derived from the TSPAN4‐EGFP^+^ OS cells.

Previous studies have suggested that migrasomes play an important role in cellular messaging, and our study confirms the involvement of OCDMs in regulating the malignant progression of OS. In BALB/C mice, OCDM treatment significantly promoted OS proliferation and lung metastasis, whereas inhibition of OS cell migrasome production significantly suppressed OS proliferation and lung metastasis, emphasizing the role of OCDMs in OS progression.

In this study, we provided evidences that OCDMs did not directly act on OS cells to affect cell proliferation, migration, or invasion. The TME is the surrounding microenvironment of tumor cells and includes blood vessels, immune cells, fibroblasts, signaling molecules, and the ECM.^[^ ^22^ ^]^ Tumors are closely related to the TME, and tumor cells can affect the TME by releasing cell signaling molecules, promoting malignant tumor progression.^[^ ^4a^ ^]^ TAMs play an important role in OS progression as a key component of the OS TME.^[^ ^6^ ^]^ Studies have revealed that exosomes derived from OS cells can promote the secretion of various pro‐tumor cytokines from TAMs by inducing M2 polarization, promoting OS progression.^[^ ^23^ ^]^ Targeting TAMs in the TME can improve the prognosis of OS patients by affecting TAM recruitment, promoting the conversion of M2 TAMs to M1 TAMs and regulating the expression of TAM immune checkpoints.^[^ ^24^ ^]^ In vivo, OCDM treatment significantly promoted M2 polarization of TAMs in OS tissues, whereas inhibition of OCDM formation in OS tissues significantly suppressed M2 polarization of TAMs. This finding suggests that OCDMs primarily alter the functions associated with TAMs in the TME. In vitro, OCDM treatment enhanced the phagocytosis of BMDMs and further promoted M2 polarization of macrophages through this effect. In addition, our results show that OCDMs also enhance THP‐1‐derived macrophages phagocytosis and promote its further M2 polarization, while have no significant effect on osteoclastogenesis (Figure S16A–P, Supporting Information). Recent study reported that sarcoma patients with more SPP1^hi^ TAMs had poorer clinical outcomes, whereas patients with more CXCL9^hi^ TAMs had better ones.^[^ ^18^ ^]^ And we found that OCDM treatment inhibited the CXCL9:SPP1 polarity of BMDMs. This finding suggested that OCDMs promote macrophage conversion to an immunosuppressive phenotype. Furthermore, in vitro experiments showed that OCDM‐treated BMDMs enhanced the proliferation, migration, and invasion capabilities of OS cells. Therefore, we revealed that OCDMs promote OS progression by regulating macrophages.

Previous studies reported that cytosolic protein can be actively translocated into migrasomes from the main body of the cell.^[^ ^10b^ ^]^ However, the mechanism underlying the packaging of cytosolic proteins into migrasomes is not clear. Our findings suggested that MANPs are functional units of the migrasomes that fulfill the role of intercellular communication between macrophages and OS cells in TME. Previous studies have demonstrated that a large number of small vesicles with diameters of 50–250 nm are formed after the breakage of cell retraction fibers; these vesicles are referred to as retractosomes. The formation of retractosomes and migrasomes depends on cell migration, and the protein composition of retractosomes is similar to that of migrasomes but distinct from that of exosomes. TSPAN4 overexpression promotes the formation of retractosomes and migrasomes, suggesting that they are closely related.^[^ ^25^ ^]^ Migrasomes are also referred to as pomegranate‐like structures because of the presence of varying numbers of internal small vesicles; however, the relationships between internal small vesicles and retractosomes and their functions remain unclear. In this study, we observed that small vesicles could be released by migrasome rupture, retraction fiber breakage and migrasome budding, and the morphology and size of small vesicles released by migrasome rupture and retraction fiber breakage were identical. Considering the relevance of small vesicles to migrasomes, we named them MANPs. In vitro experiments showed that, similar to migrasomes, MANP treatment similarly enhanced the phagocytosis of BMDMs, promoted M2 polarization and inhibited CXCL9:SPP1 polarity, suggesting a functional correlation between MANPs and migrasomes. Previous studies have revealed a positive correlation between the number of small vesicles inside the migrasome and the diameter of the migrasome, and migrasome formation can be divided into two phases: the first phase, in which the retraction fibers locally expand to form young migrasomes lacking TSPAN4; and the second phase, in which TSPAN4 is enriched, where young migrasomes rapidly expand and transform into migrasomes.^[^ ^11^, ^13^ ^]^ In this study, we observed that OS cells can produce a large number of migrasomes with diameters smaller than 0.5 µm located at the ends or bifurcations of retraction fibers and that purified migrasomes with diameters of ≈0.5 µm lack internal small vesicles, suggesting that young migrasomes may lack these small vesicles. Purification of young migrasomes with diameters of 0.22–0.45 µm revealed that the number of internal small vesicles was significantly lower than that of migrasomes with diameters of 0.5–3 µm, and proteome sequencing analysis revealed that the expression level of TSPAN4 was significantly greater in MANPs than in migrasomes (Figure S17, Supporting Information). TSPAN4 is recruited to migrasomes during the growth phase while stops after a migrasome enters the steady phase.^[^ ^11a^ ^]^ Therefore, we speculate that MANPs influx may lead to the rapid enrichment of TSPAN4 in young migrasomes, thus promoting young migrasome maturation until migrasome enters the steady phase, explaining the correlation between migrasome diameter and the number of internal vesicles. Previous studies have demonstrated that migrasomes survive for only ≈400 min and readily rupture and release their contents upon maturation or detachment from cells.^[^ ^10b^ ^]^ Therefore, we hypothesize that in organisms, MANPs with an intact membrane structure are the main functional unit of the migrasome messaging function and that their smaller size may allow them to travel through the circulatory system to exercise their function in locations that migrasomes cannot reach. In this study, we found that compared with migrasomes, young migrasomes lacking MANPs demonstrated a significantly reduced ability to enhance BMDM phagocytosis, promote M2 polarization and inhibit CXCL9:SPP1 polarity. This finding suggests that MANPs are functional units of the migrasomes that fulfill the role of intercellular communication between macrophages and OS cells in TME.

We identified MFGE8 as a key protein in OCDMs and MANPs that plays a regulatory role in promoting OS progression. Our proteome sequencing results showed that MFGE8 is significantly enriched in OCDMs and MANPs. In addition, our results show that MFGE8 and TSPAN4 are highly expressed in OS tissues compared to corresponding non‐tumor tissues, and are highly expressed in lung metastatic tissues compared to corresponding tumor tissues (Figures S18A–C and S19A–C, Supporting Information). Besides, MFGE8 and TSPAN4 are positively correlated with macrophage M2 polarization marker in OS samples (Figure S20A,B, Supporting Information). MFGE8 is a secreted multifunctional glycoprotein that plays an important role in tumor metastasis, myocardial injury repair, and fatty liver treatment.^[^ ^21^, ^26^ ^]^ Previous studies have shown that vertebral skeletal stem cells promote the preferential metastasis of breast, prostate, and lung cancer cells to vertebrae but not other bones through the specific secretion of MFGE8.^[^ ^26a^ ^]^ MFGE8‐enriched MSC‐derived exosomes enhance macrophage phagocytosis of apoptotic cardiomyocytes and M2 polarization to promote cardiac recovery after myocardial infarction.^[^ ^21^ ^]^ We found that the promotion of macrophage phagocytosis and M2 polarization and inhibition of CXCL9:SPP1 polarity by OCDMs and MANPs was significantly attenuated after the knockdown of MFGE8 in OCDMs and MANPs in vitro. This finding suggested that OCDMs and MANPs regulate macrophage function mainly by enriching MFGE8. In vivo, the promotion of OS proliferation and lung metastasis by OCDMs was significantly attenuated after the knockdown of MFGE8 in OCDMs. This finding suggests that OCDMs regulate macrophage function, thus promoting OS progression mainly by enriching MFGE8.

## 4 Conclusion

**Figure 8:**
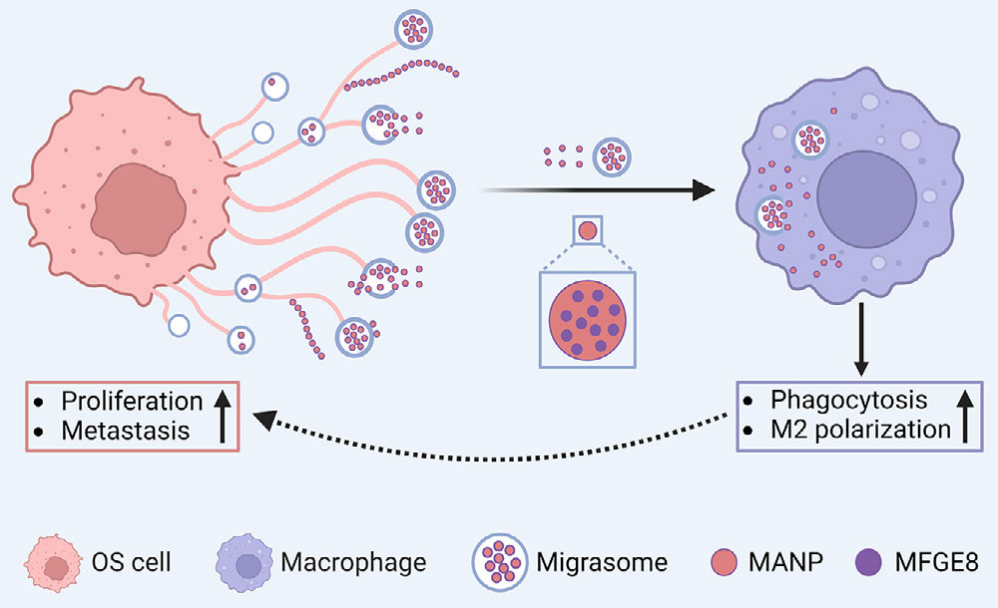
Schematic illustration of the OCDMs promote OS progression. OCDMs enhance M2 polarization of macrophage of tumor microenvironment (TME) to aggravate OS progression via MFGE8 (created with BioRender.com).

In summary, our results reveal the potential role of OCDMs in OS progression. OCDMs enhance macrophage phagocytosis and promote macrophage M2 polarization, promoting OS proliferation and lung metastasis. MANPs are the key functional units through which OCDMs communicate between OS cells and macrophages, and MFGE8 is a key protein through which OCDMs and MANPs exert their regulatory effects on macrophages (**Figure** **8**). Our findings indicate that targeting OCDMs may have high OS therapeutic potential by regulating the osteosarcoma TME and inhibiting OS progression.

## 5 Experimental Section

### Animal Experiments

Four‐week‐old female BALB/c mice were purchased from Zhejiang Vital River Laboratory Animal Technology Co., Ltd., and housed in the Laboratory Animal Centre of Nanjing First Hospital (Animal Use License: SYXK 2021‐0007). Mice were acclimated for one week before the experiment. To test the effect of migrasomes on OS progression, twelve BALB/c mice were randomly divided into two groups, and 10 µL of 5 × 10^5^ OS cells was injected into the proximal right tibia of each mouse. After 14 days, two groups of mice were injected intratumorally with 50 µg of migrasome or an equal volume of PBS every 3 days. To test the effect of inhibiting OCDM formation on OS progression, eighteen BALB/c mice were randomly divided into three groups. The first group was injected with 10 µL of 5 × 10^5^ sh‐NC OS cells in the proximal right tibia, and the second and third groups were injected with 10 µL of 5 × 10^5^ sh‐TSPAN4 OS cells in the proximal right tibia. After 14 days, three groups of mice were injected intratumorally with 50 µg of migrasome or an equal volume of PBS every 3 days. To detect the effect of MFGE8 knockdown in OCDM on OS progression, twelve BALB/c mice were randomly divided into two groups, and 10 µL of 5 × 10^5^ OS cells was injected into the proximal right tibia of each group of mice. After 14 days, two groups of mice were injected intratumorally with 50 µg of sh‐NC‐migrasomes or 50 µg of sh‐MFGE8‐migrasomes every 3 days. Tumor volume was calculated as follows: volume = 0.5 × length × (width)^2^. Twenty‐eight days after OS cell implantation, the tumor tissues were weighed and subjected to flow cytometry and immunohistochemistry, and HE staining was performed on the lung tissues. To test the presence of OS‐derived migrasomes and MANPs in vivo, 10 µL of 5 × 10^5^ TSPAN4‐EGFP‐OE OS cells was injected into the proximal right tibia of three BALB/c mouse, respectively. After 28 days, the tumor tissues were subjected to immunofluorescence staining.

### Flow Cytometric Analysis

To assess immune cell changes in OS tissues, tumors were minced and digested with a digest containing 0.5 mg mL^−1^ DNase and 1 mg mL^−1^ type IV collagenase for 1 h at 37 °C, filtered through a 70 µm cell sieve, washed with PBS and incubated with an anti‐mouse CD16/32 antibody (14‐0161, eBioscience, USA) for 15 min at 4 °C on ice. Fixable viability dye (65‐0865, eBioscience, USA) was incubated for half an hour, and the cells were washed in PBS and labeled with the following antibodies: anti‐CD45 (56‐0451, eBioscience, USA), anti‐CD11b (69‐0112, eBioscience, USA), anti‐CD49b (17‐5971, eBioscience, USA), anti‐Ly‐6G (367‐9668, eBioscience, USA), anti‐CD3 (46‐0032, eBioscience, USA), anti‐CD4 (48‐0041, eBioscience, USA), anti‐CD8 (11‐0081, eBioscience, USA), anti‐CD25 (61‐0251, eBioscience, USA), anti‐F4/80 (17‐4801, eBioscience, USA), anti‐CD86 (46‐0862, eBioscience, USA) and anti‐CD206 (12‐2061, eBioscience, USA). Then cells were fixed, permeabilized, and then labeled with anti‐FOXP3 (12‐5773, eBioscience, USA). The cells were then washed with PBS, and flow assays were performed using a flow cytometer (FACSymphony A5 SORP, BD Biosciences, USA).

To assess the level of apoptosis in OS cells, it induced apoptosis using an apoptosis induction kit (C0005, Beyotime, China) followed by treatment with an Annexin V‐FITC/PI Apoptosis Detection Kit (KGA1102, KeyGEN, China). Flow cytometry assays were performed using a flow cytometer (FACSCalibur, BD Biosciences, USA).

To assess the ability of macrophages to phagocytose apoptotic OS cells, apoptotic OS cells were labeled with CFSE (M5117, AbMole, USA), and after cocultivation of macrophages with apoptotic OS cells for 1 h, after labeling macrophages with an anti‐F4/80 flow cytometry antibody, flow cytometry assays were performed using a flow cytometer (FongCyte, Beijing Challen Biotechnology Co., Ltd., China).

To assess the M1 and M2 polarization ability of BMDMs, flow cytometry assays were performed after the BMDMs were labeled with anti‐F4/80, anti‐CD86 and anti‐CD206 antibodies. All the data were analyzed using FLowJo‐v10.8.1 flow cytometry analysis software (TreeStar, USA).

### Cell Lines

The human OS cell lines 143b and MG63 and the mouse OS cell line K7M2 wt were obtained from the American Type Culture Collection (ATCC), and the OS cell lines were cultured in Dulbecco's modified Eagle's medium (DMEM, C11995500BT, Gibco, USA) supplemented with 1% penicillin/streptomycin (P/S, 15140122, Gibco, USA) and 10% fetal bovine serum (FBS, 10091148, Gibco, USA). RAW 264.7 cells were obtained from ATCC and cultured in DMEM supplemented with 1% P/S and 10% FBS, after stimulated with 100ng mL^−1^ receptor activator of nuclear factor‐κB ligand (RANKL, 50343‐M01H, SinoBiological, China) for 5 days, RAW 264.7 cells were differentiated to osteoclasts and fixed and stained using the TRAP staining kit (387A, Sigma‐Aldrich, USA). THP‐1 cells were obtained from ATCC and cultured in Roswell Park Memorial Institute (RPMI) 1640 medium (KGL1501‐500, KeyGEN, China) supplemented with 10% FBS, 0.05 mM β‐mercaptoethanol (PB180633, Procell, China) and 1%PS, after incubated with 100 ng mL^−1^ phorbol 12‐myristate 13‐acetate (PMA, P1585, Sigma‐Aldrich, USA) for 2 days, THP‐1 cells were differentiated to M0 macrophages. All cells were cultured in an incubator with 5% CO2 at 37 °C.

### Primary Mouse BMDM Culture

Cells were isolated from the tibia and femur of 8‐week‐old BALB/c mice and cultured in DMEM supplemented with 10% FBS, 1% PS, and 20 ng mL^−1^ M‐CSF (315‐02‐50, PeproTech, USA) for 6 days to allow differentiation into macrophages.

### Lentiviral Infection

Lentiviral vectors containing sh‐TSPAN4 and sh‐MFGE8 coding sequences together with corresponding negative controls were obtained from GenePharma, and stably infected K7M2 wt cells were selected with puromycin (ST551, Beyotime, China). Lentiviral vectors containing TSPAN4‐EGFP‐OE together with corresponding negative controls were obtained from KeyGEN (China), and stably infected K7M2 wt cells were selected with puromycin. qRT–PCR and WB were used to assess the efficiency of lentivirus infection.

### TEM

Fresh tumor tissue was cut into tissue blocks of less than 1 mm^3^ with a blade, placed in EP tubes, fixed with a special fixative for electron microscopy for 2 h, and stored and transported at 4 °C. The migrasomes and MANPs were processed using the standard TEM sample preparation and observed by TEM (JEM‐1400, JEOL, Japan).

Purified migrasomes and young migrasomes were precipitated, fixed, and subjected to the standard sample preparation process for TEM, and the internal structure was observed by TEM (Tecnai G2, FEI, USA). Purified migrasomes, young migrasomes, and MANPs were negatively stained with uranyl acetate, and the structures were observed by TEM.

### SEM

OS cells were inoculated onto glass slides in 24‐well plates at a density of 40%, cultured for 16 h, and fixed with a special fixative for electron microscopy. After a standard sample preparation process for SEM, the migrasomes and young migrasomes of OS cells were observed via SEM (JSM‐7900F, JEOL, Japan), and the MANPs of OS cells were observed via SEM (Regulus 8100, Hitachi, Japan).

### Immunofluorescence Staining

To observe OS‐derived migrasomes and MANPs in OS tissues, it cut tumor tissue samples into sections (4 µm) in which OS cells stably expressing TSPAN4‐EGFP fluorescence, fixed them with 4% paraformaldehyde for 15 min, permeabilized them with 0.3% Triton‐X100 for 15 min, blocked them with 3% BSA for 1 h, and incubated them with a RAB8a (1:500, ab188574, Abcam) antibody overnight, after which the sections were incubated with secondary antibody for 1 h at room temperature. Then, images were captured using a confocal microscopy.

To observe the migrasomes, young migrasome, and MANPs of OS cells, it inoculated OS cells onto glass slides at a density of 40% for 16 h. After 16 h, the cells were fixed with 2.5% glutaraldehyde fixative for 15 min, washed with PBS, and stained with 1 µg mL^−1^ WGA (W11261, Invitrogen, USA) for 15 min. Migrasomes were observed with a confocal microscope (Lsm880 NLO, Zeiss, Germany), whereas young migrasomes and MANPs were observed with a superresolution microscope (Multi‐SIM, NanoInsights/Zeiss).

OS cells were inoculated at a density of 40% and cultured in a confocal dish for 16 h to observe the release of MANPs from OS cell migrasomes. The OS cells were then stained with 1 µg mL^−1^ WGA, and the confocal dish was placed under a superresolution microscope. Humidity, a temperature of 37 °C, and 5% CO2 were maintained, and images were acquired every 15 min.

OS cells were inoculated onto glass slides at a density of 40% for 16 h to observe the localization of MFGE8 in OS cells. OS cells were fixed with 4% paraformaldehyde for 30 min, permeabilized with 0.3% Triton‐X100 for 15 min, blocked with 3% BSA for 1 h, incubated with an anti‐MFGE8 (1:200, PA5‐109955, Invitrogen, USA) antibody dilution overnight, incubated with secondary antibody for 1 h at room temperature, and stained with 1 µg mL^−1^ WGA for 15 min before observation by confocal microscopy.

WGA‐labeled migrasomes or Dil‐labeled MANPs and young migrasomes were added to the culture dish after the inoculation of macrophages into the confocal culture dish to observe the uptake of migrasomes, young migrasomes, and MANPs by macrophages. After 24 h of coincubation, the cells were fixed with 4% paraformaldehyde for 30 min, and the nuclei were stained with DAPI and observed by confocal microscopy.

To assess the effect of SAR407899 on OCDMs, it inoculated OS cells onto glass slides at a density of 40%. After treated with DMSO or 10 µM SAR407899 (HY‐15687A, MCE, USA), the cells were fixed with 2.5% glutaraldehyde fixative for 15 min, washed with PBS, and stained with 1 µg mL^−1^ WGA for 15 min. Migrasomes were observed with a confocal microscope.

To assess BMDMs phagocytosis, it incubated BMDMs with 2 µm diameter FITC‐labeled microbeads for 1 h, washed them with PBS to remove free microbeads, fixed them with 4% paraformaldehyde for 30 min, permeabilized them with 0.3% Triton‐X100 for 15 min, blocked them with 3% BSA for 1 h, and incubated them with diluted anti‐F4/80 (14‐4801‐82, Invitrogen, USA) antibodies overnight; the BMDMs were subsequently incubated with secondary antibodies for 1 h at room temperature. Images were captured by fluorescence microscopy. Immunofluorescence images were analyzed with ZEN software (Zeiss, Germany), and 3D reconstruction of the images was performed using Imaris 9.0.1 software.

### Migrasome, MANP, and Young Migrasome Purification

OS cells were inoculated into 150 mm dishes and cultured in conditioned medium containing exosome‐free serum (EXO‐FBS‐50A‐1, Thermo Fisher, USA). When the cells reached 40–50% confluence, they were washed twice with PBS, digested with 0.25% trypsin, and collected in 50 ml centrifuge tubes. All subsequent operations were performed at 4 °C. The samples were centrifuged at 1000 × g for 10 min and 4000 × g for 20 min to remove cells and large quantities of cellular debris. The collected supernatant was filtered through a 0.45 µm filter and then a 0.22 µm filter. The filtrate was transferred to a new tube and centrifuged at high speed for 5 min at 18 000 × g. The supernatant was aspirated and washed with PBS, and the MANP was collected by centrifugation at 2000 × g for 5 min in a 100 kDa ultrafiltration centrifuge tube (UFC810024, Millipore, USA). To isolate migrasomes or young migrasomes, 0.45 µm or 0.22 µm filters were reversed and squeezed with a medical syringe containing PBS. The filtrate was centrifuged at 2000 × g for 5 min in a 100 kDa ultrafiltration tube to collect the migrasomes or young migrasomes. The expression of migrasome signature proteins in the migrasomes and MANPs was detected by WB, the morphology of the migrasomes, MANPs, and young migrasomes was observed by TEM, and the particle diameter of the MANPs was measured using a nanoparticle tracking analyzer (ZetaVIEW, PARTICLE METRIX).

### CCK‐8 and EdU Assays

These functional in vitro experiments for testing cell proliferation in OS cells were conducted as described in previous studies.^[^ ^27^ ^]^

### Transwell Assays

Transwell assays were conducted to determine the impacts on cell invasion and migration as previously described.^[^ ^27^ ^]^

### Cell Coculture

To assess the effect of BMDMs on OS cells, it inoculated OS cells into 6‐well plates and BMDMs into the upper chamber of 0.4 µm coculture chambers (MCHT06H48, Millipore, USA). The coculture chambers were inserted into the 6‐well plates. After 48 h of coculture, OS cell proliferation, migration, and invasion were assayed.

### qRT‐PCR

RNA‐Easy Isolation Reagent (R701, Vazyme, China) was used to extract total RNA from tissues and cells. HiScript II Q RT SuperMix for qPCR (R223, Vazyme, China) was used to reverse transcribe the mRNA according to the manufacturer's instructions. The ChamQ SYBR qPCR Master Mix (Q321, Vazyme, China) was then used to conduct qPCR on a LightCycler 96 (Roche, Switzerland), and β‐actin was used as the internal control. All primer sequences are displayed in Table S1 (Supporting Information).

### WB

WB was routinely conducted according to the previous reports.^[^ ^28^ ^]^ Briefly, total protein was extracted from tissues, cells and vesicles using RIPA lysis buffer for WB analysis. The following primary antibodies were applied: PIGK (1:500, sc‐398611, Santa Cruz Biotechnology), CPQ (1:1000, 16601‐1‐AP, Proteintech), NDST1 (1:1000, sc‐374529, Santa Cruz Biotechnology), GAPDH (1:1000, 5174, CST), TSPAN4 (1:1000, A10253, ABclonal), MFGE8 (1:1000, PA5‐109955, Thermo Fisher), and β‐actin (1:1000, 8457, CST). The signals of specific proteins were detected with a Tanon 5200 automatic chemiluminescence imaging analysis system (Shanghai, China).

### Transcriptome and Proteome Sequencing

Using the Illumina NovaSeq 6000 sequencing platform, BMDMs treated with PBS or migrasomes (*n* = 3/group) were subjected to transcriptome (RNA‐seq) analysis, differential gene expression analysis, and differential gene function enrichment analysis; |FoldChange| >2 and padj <0.05 were used as the screening criteria for DEGs. The cell body, migrasomes, and MANPs (n = 3/group) were sequenced using 4D label‐free quantitative proteomics technology on a tims‐TOF Pro mass spectrometer; |FoldChange| >2 and padj <0.05 were used as the screening criteria for differentially expressed proteins, and the identified proteins were systematically analyzed by bioinformatics.

### IHC

After antigen retrieval and blocking, tissue sections (4 µm) were incubated with primary antibodies against Ki‐67 (1:200, 12202, CST), vimentin (1:200, 5741, CST), E‐cadherin (1:400, 3195, CST), CD86 (1:200, 19589, CST), and CD206 (1:200, 24595, CST) overnight at 4 °C and then with secondary antibodies for 1 h at room temperature. Sections were treated with diaminobenzidine for staining. Then, the sections were photographed using a Nikon microscope, and protein expression levels were quantified using ImageJ software (Loci, USA).

### Clinical Specimens

OS tissues and matches of non‐tumor normal tissues were collected from patients at the Nanjing First Hospital. The OS tissue specimens were quickly frozen in liquid nitrogen and stored at −80 °C until protein and RNA extraction. The study was approved by the Ethics Committee of the Nanjing First Hospital of Nanjing Medical University, and written informed consent was obtained from all patients.

### Microarray Data

To assess the correlation between TSPAN4/MFGE8 and CD163 expression in OS, gene‐expression profile (GEO: GSE12865) of OS were downloaded from the GEO database (https://www.ncbi.nlm.nih.gov/geo/). Correlation analysis was conducted by the Spearman method and the ggplot2 package was used to visualize the results. All of the above analyses were performed using R (version 4.2.1).

### Statistical Analysis

Statistical analysis and graphic presentation were performed using GraphPad Prism version 8 (GraphPad Software, USA). The normality of data was tested by Kolmogorov–Smirnov test. Unpaired two‐tailed Student's t‐test was performed for the comparison between two groups. One‐way ANOVA test or two‐way ANOVA test was used for multivariate analysis. Data were shown as mean ± SD and all statistical analyses were based on a minimum of three samples per group. *P*‐values < 0.05 were considered as statistically significant. The detailed statistical analysis and sample size applied to each experiment was presented in the corresponding figure legends.

### Ethical Statement

All animal experiments were performed according to the protocol approved by the Experimental Animal Ethics Committee of Nanjing First Hospital (Approval No. DWSY‐23021325). The Ethics Committee of the Nanjing First Hospital of Nanjing Medical University approved our study (Approval No. KY20221124‐04).

## Conflict of Interest

The authors declare no conflict of interest.

## Author Contributions

W.L. and L.L. contribute equally to the study. W.L. designed and performed the experiments, collected and analyzed the data, and drafted the manuscript. L.L. contributed to the experimental design and revised the manuscript. X.B., M.Z., W.L. contributed to the experimental design and the manuscript. Y.M. and Y.S. performed the animal experiments and collected the data. H.Z. contributed to the experimental design and the manuscript. Q.J., Q.Y., and Z.Z. designed and supervised the study and critically revised the manuscript. All authors have read and approved the final manuscript.

## Supporting information



Supporting Information

## Data Availability

The data that support the findings of this study are available from the corresponding author upon reasonable request.
