# Supplementary material for: Osteosarcoma Cell‐Derived Migrasomes Promote Macrophage M2 Polarization to Aggravate Osteosarcoma Proliferation and Metastasis
Source: Adv Sci (Weinh). 2025 Mar 8;12(17):2409870. doi: 10.1002/advs.202409870 (PMC12061288; doi:10.1002/advs.202409870)
Supplement: Supplementary file 1 — Supporting Information [file ADVS-12-2409870-s001.docx]

Supporting Information

Osteosarcoma Cell-Derived Migrasomes Promote Macrophage M2 Polarization to Aggravate Osteosarcoma Proliferation and Metastasis

Wanshun Liu, Lei Li, Xiaoming Bai, Mengxue Zhang, Wei Lv, Yongbin Ma, Yuzhi Sun, Hongjing Zhang, Qing Jiang*, Qingqiang Yao*, Zhi-Yuan Zhang*


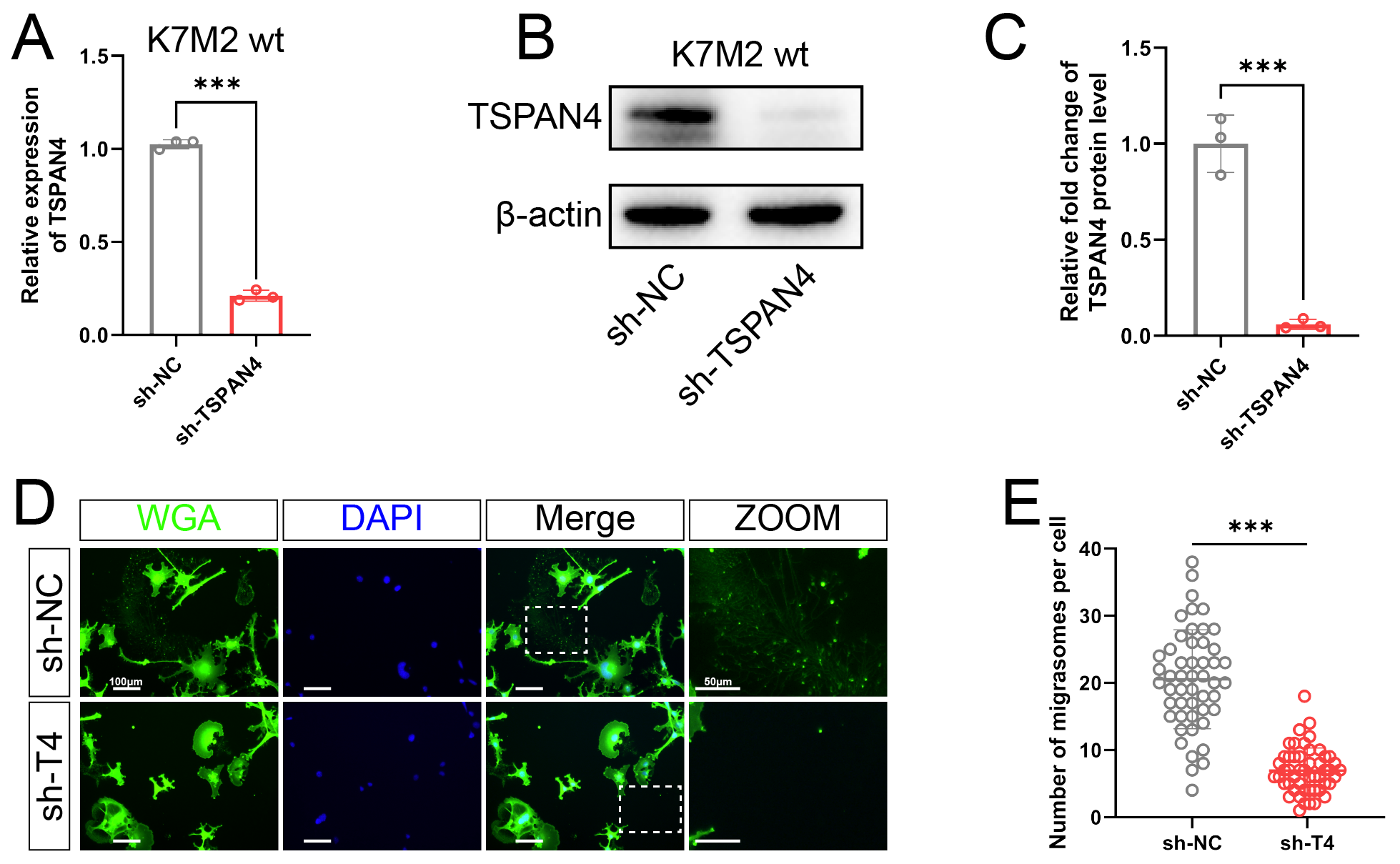


**Figure S1.** **Knockdown of TSPAN4 inhibits the formation of migrasomes in OS cells.**

(A) RT-qPCR analysis of TSPAN4 expression after transfection in K7M2 wt cells (*n* = 3). (B, C) Western blot analysis of TSPAN4 expression after transfection in K7M2 wt cells and quantitative analysis (*n* = 3). (D, E) Representative immunostaining images of WGA (green) and DAPI (blue) in K7M2 wt cells and quantitative analysis of migrasomes per cell (*n* = 50). Low magnification: scale bars = 100 µm; high magnification: scale bars = 50 µm. Results were shown as mean ± SD. ****p* < 0.001. Unpaired t-tests were used for the comparison of two groups.


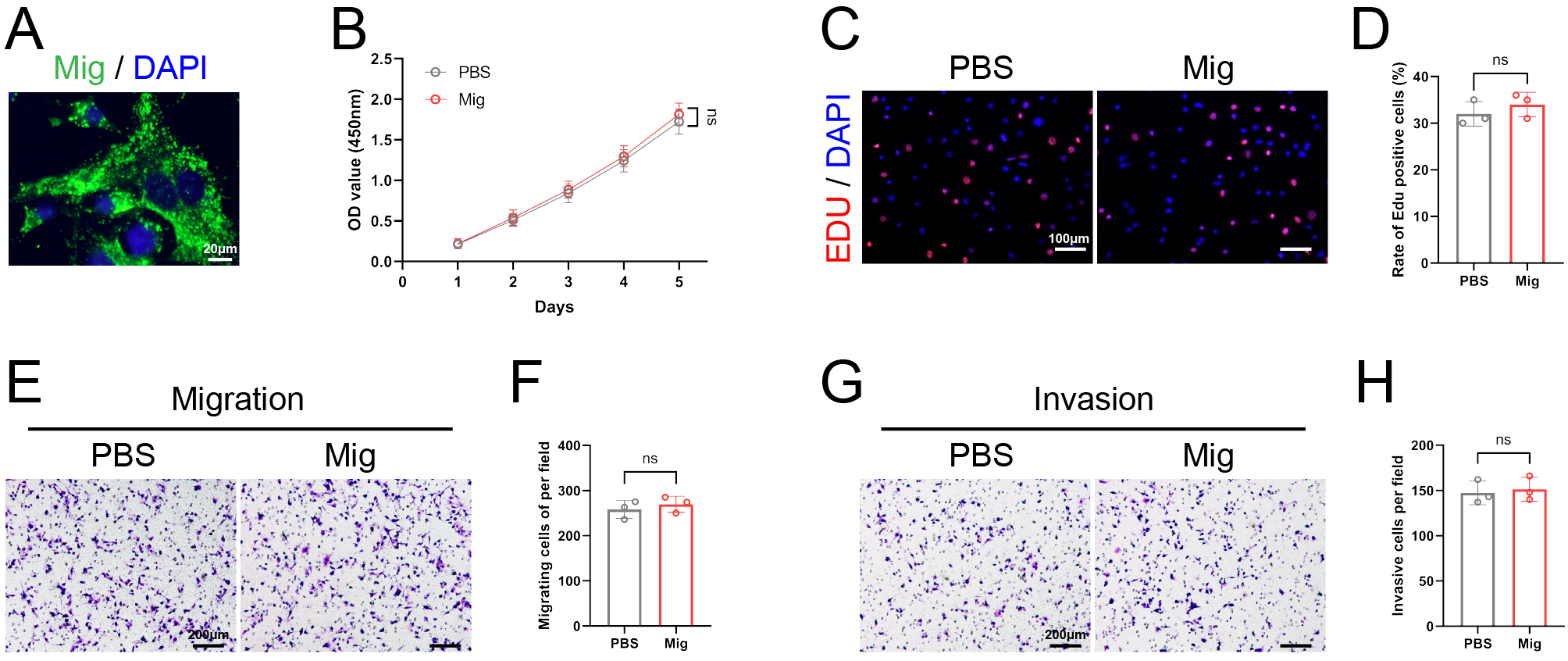


**Figure S2.** **Effect of OCDMs on OS cell proliferation, migration and invasion *in vitro*.**

(A) Representative immunostaining images of K7M2 wt cells phagocytosis of WGA-labeled migrasomes (green) (*n* = 3). Scale bars = 20 µm. (B-H) K7M2 wt cells treated with PBS or 10μg/ml migrasomes for 24 hours. (B) CCK8 analysis of OS cells (*n* = 3). (C, D) Representative images and quantitative analysis of OS cells EDU assays (*n* = 3). Scale bars = 100 µm. (E, F) Representative images and quantitative analysis of OS cells transwell migration assays (*n* = 3). Scale bars = 200 µm. (G, H) Representative images and quantitative analysis of OS cells transwell invasion assays (*n* = 3). Scale bars = 200 µm. Results were shown as mean ± SD. ^ns^*p* ≥ 0.05. One-way ANOVA test or two-way ANOVA test was used for multivariate analysis. Unpaired t-tests were used for the comparison of two groups.


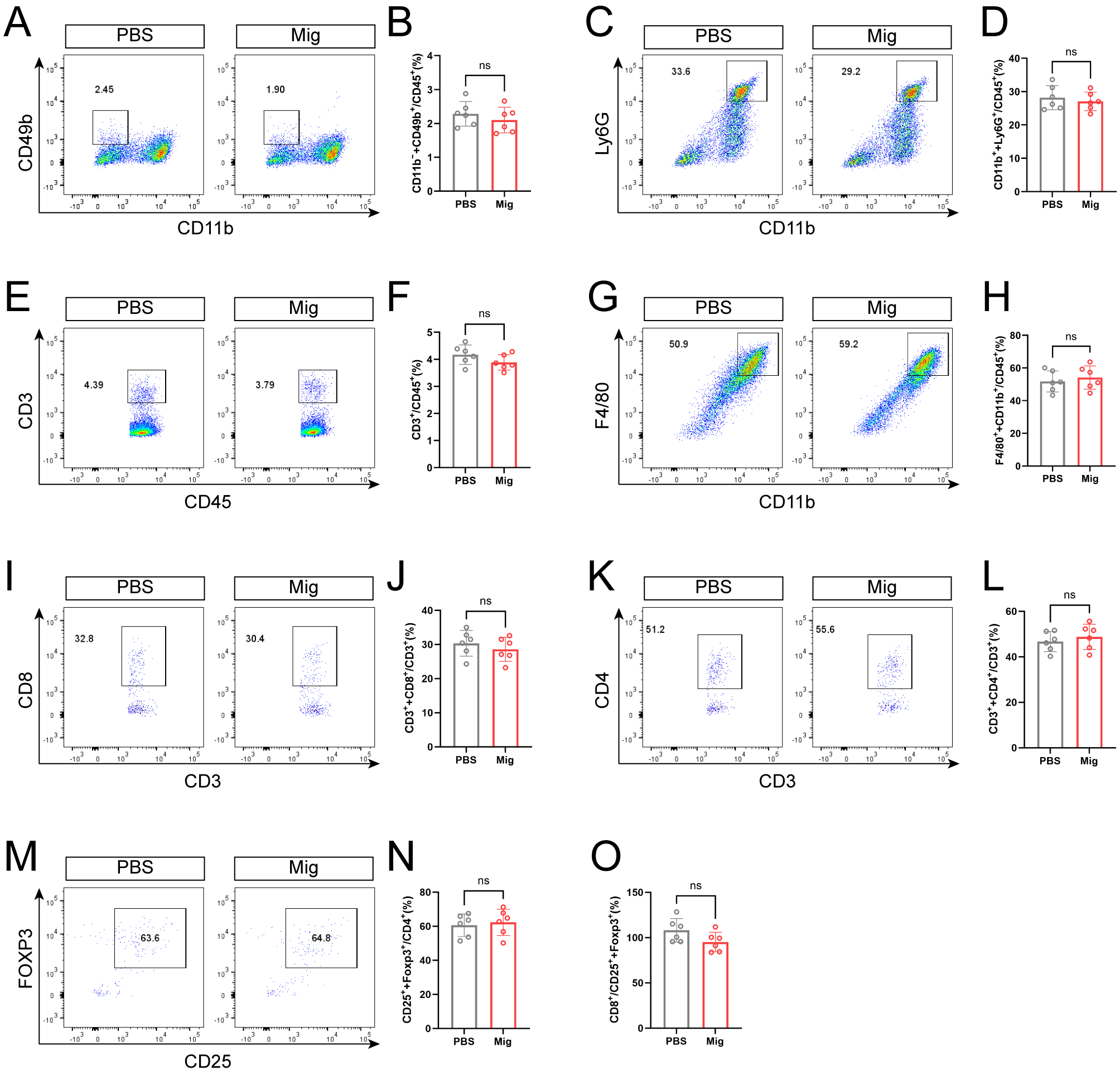


**Figure S3.** **Effect of OCDMs on the immune microenvironment of OS.**

(A, B) Flow cytometry analysis of NK cell ratio in tumors and quantitative analysis (*n* = 6). (C, D) Flow cytometry analysis of neutrophil ratio in tumors and quantitative analysis (*n* = 6). (E, F) Flow cytometry analysis of T cell ratio in tumors and quantitative analysis (*n* = 6). (G, H) Flow cytometry analysis of macrophage ratio in tumors and quantitative analysis (*n* = 6). (I-J) Flow cytometry analysis of CD8^+^ T cells ratio in T cells and quantitative analysis (*n* = 6). (K-L) Flow cytometry analysis of CD4^+^ T cells ratio in T cells and quantitative analysis (*n* = 6). (M-N) Flow cytometry analysis of Tregs ratio in CD4^+^ T cells and quantitative analysis (*n* = 6). (O) Quantitative analysis of the CD8/Treg ratio (*n* = 6). Results were shown as mean ± SD. ^ns^*p* ≥ 0.05. Unpaired t-tests were used for the comparison of two groups.


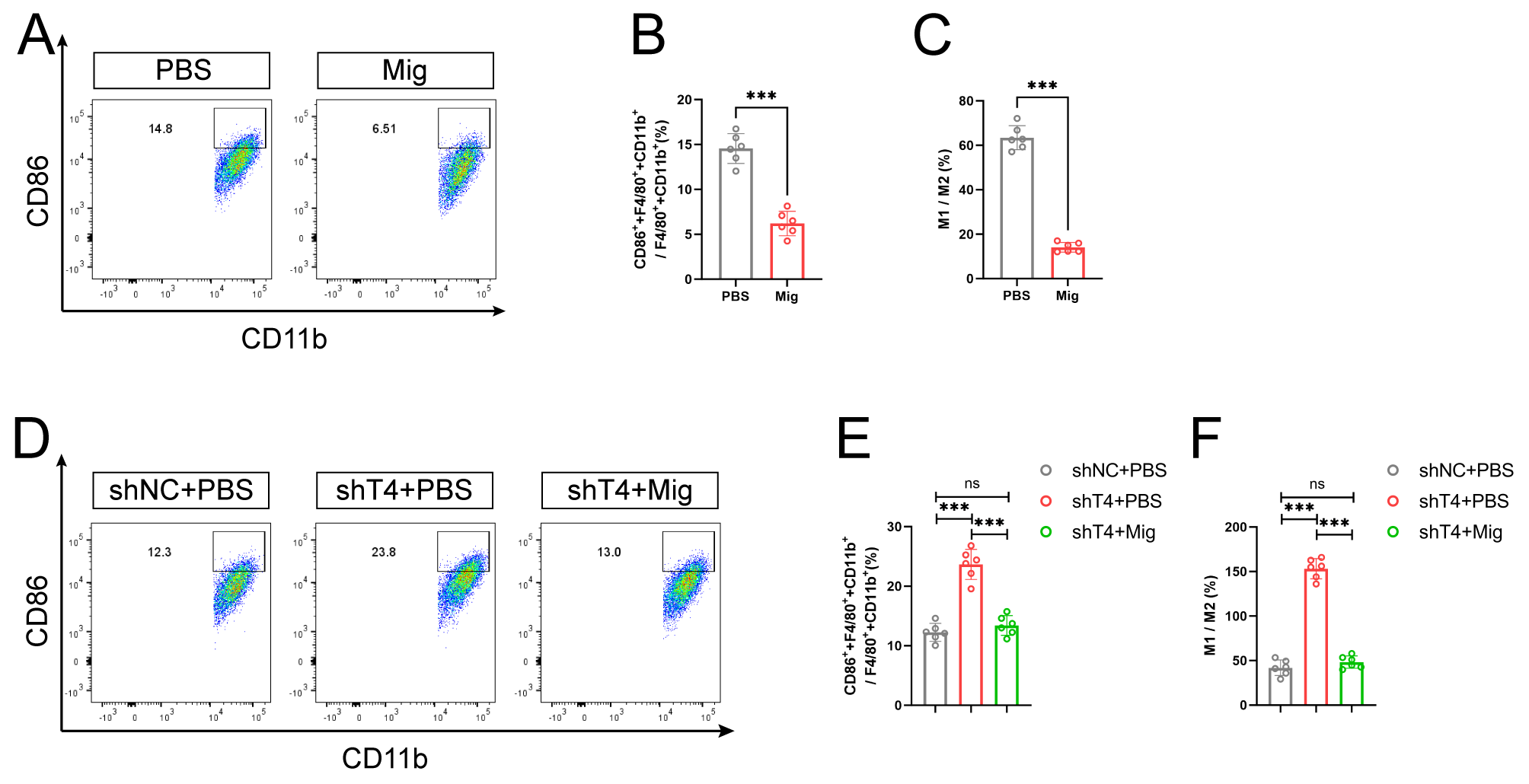


**Figure S4.** **Effect of OCDMs on macrophages M1 polarization *in vivo*.**

(A-C) After tibia injection of OS cells, mice were treated with PBS or migrasomes. Flow cytometry analysis of the proportion of M1-type macrophages in tumor tissues and quantitative analysis (*n* = 6). (D-F) After tibia injection of TSPAN4 knockdown or negative control OS cells, mice were treated with PBS or migrasomes. Flow cytometry analysis of the proportion of M1-type macrophages in tumor tissues and quantitative analysis (*n* = 6). Results were shown as mean ± SD. ^ns^*p* ≥ 0.05, ****p* < 0.001. One-way ANOVA test or two-way ANOVA test was used for multivariate analysis. Unpaired t-tests were used for the comparison of two groups.


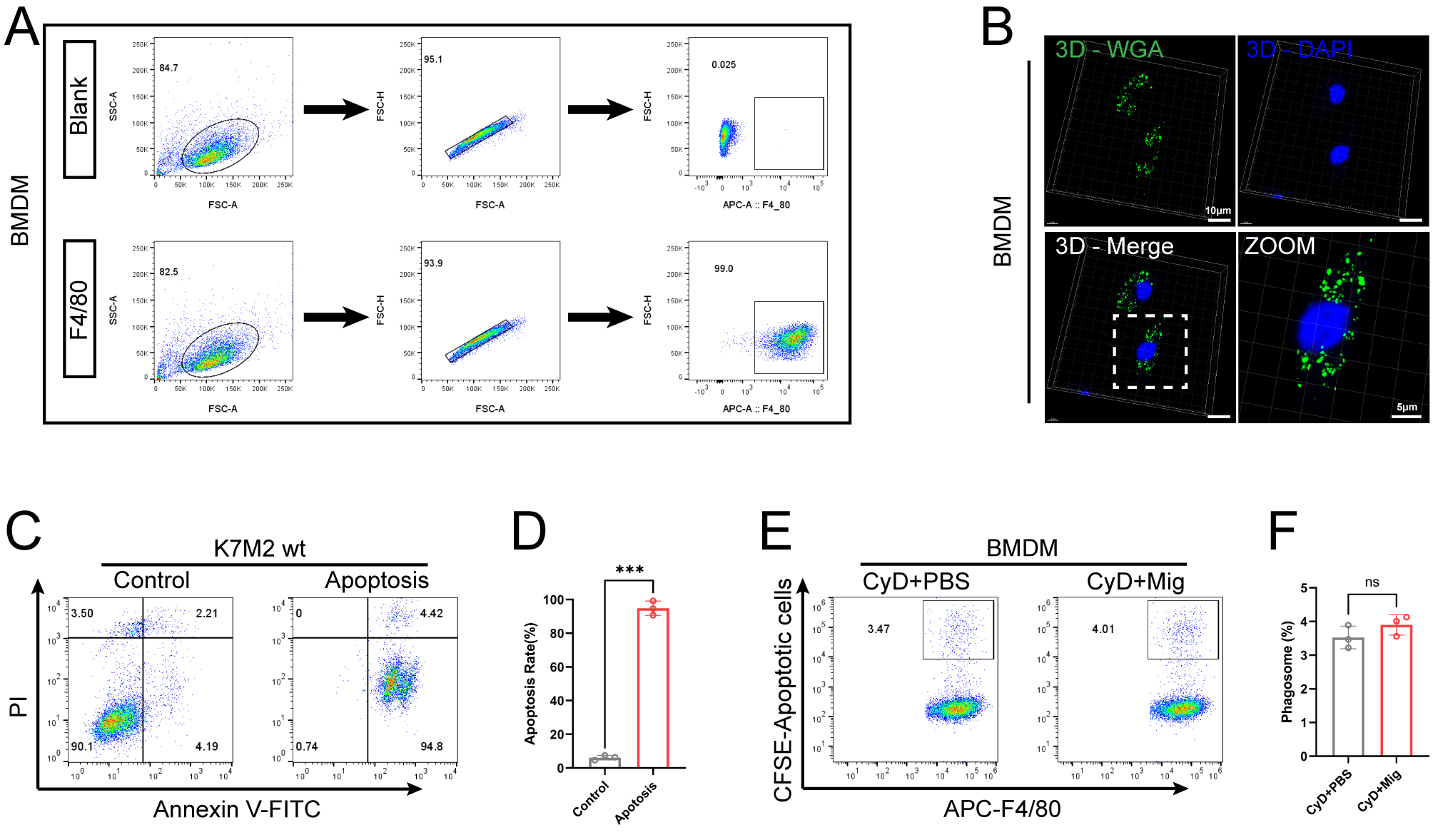


**Figure S5.** **Effect of cytochalasin D treatment on phagocytosis of BMDMs.**

(A) Flow cytometry analysis of macrophage proportions (*n* = 3). (B) Representative immunostaining images of BMDMs phagocytosis of WGA-labeled migrasomes (green) (*n* = 3). Low magnification: scale bars = 10 µm; high magnification: scale bars = 5 µm. (C, D) Flow cytometry analysis of the proportion of apoptotic OS cells and quantitative analysis (*n* = 3). (E, F) BMDMs were treated with cytochalasin D (CyD, 10 μM) overnight, followed by treatment with PBS or 10μg/ml migrasomes for 24 h, and after co-incubation with apoptotic OS cells for 1 h, Flow cytometry analysis of phagocytose apoptotic OS cells (CFSE) in BMDMs (APC) and quantitative analysis (*n* = 3). Results were shown as mean ± SD. ^ns^*p* ≥ 0.05, ****p* < 0.001. Unpaired t-tests were used for the comparison of two groups.


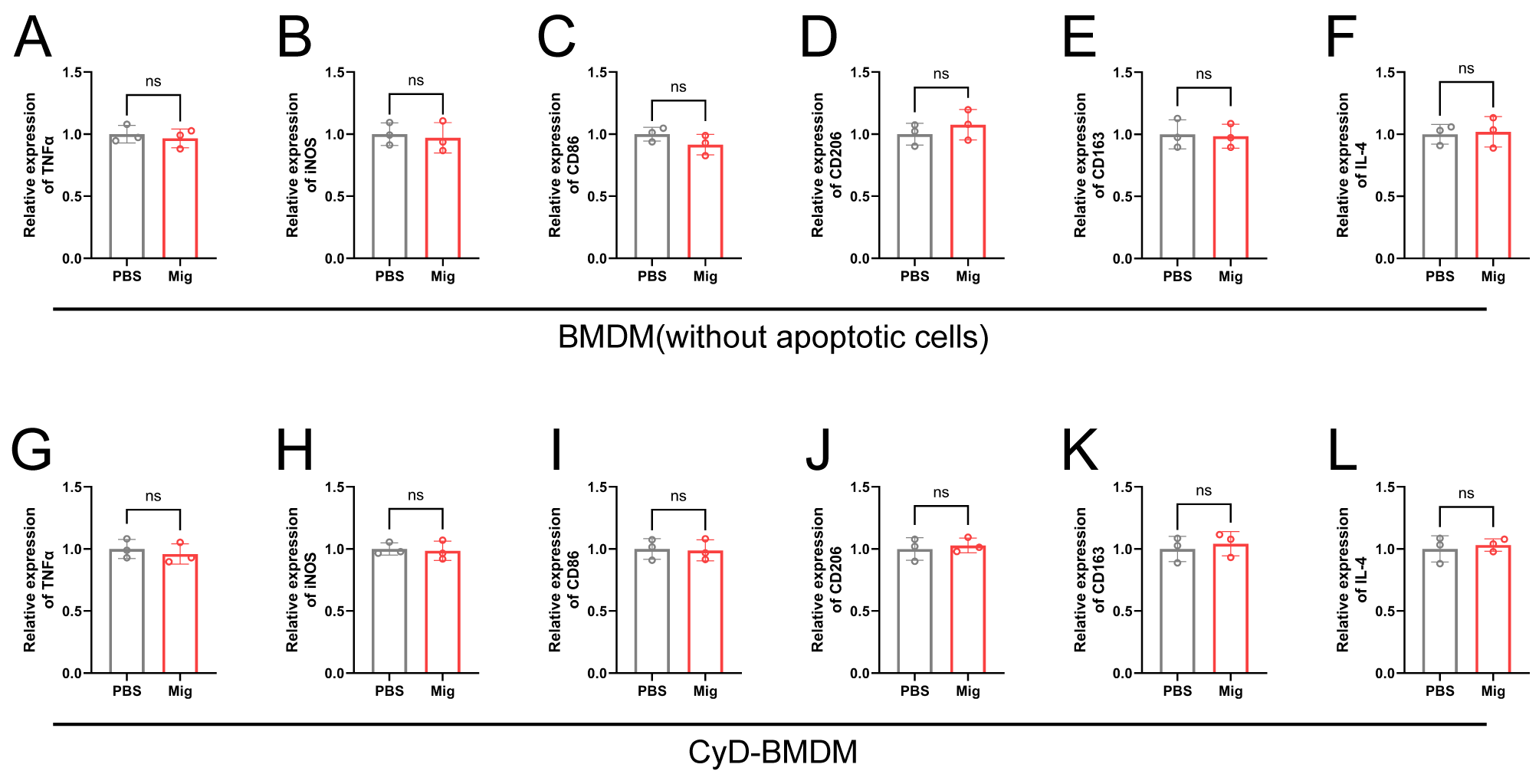


**Figure S6.** **OCDMs promote M2 polarization by enhancing macrophage phagocytosis.**

(A-F) BMDMs treated with 10μg/ml migrasomes or PBS for 24 hours. RT-qPCR analysis of the expression of M1 polarization and M2 polarization markers in BMDMs (*n* = 3). (G-L) BMDMs were treated with CyD (10 μM) overnight, followed by treatment with PBS or 10μg/ml migrasomes for 24 h, and after co-incubation with apoptotic OS cells for 24 h, RT-qPCR analysis of the expression of M1 polarization and M2 polarization markers in BMDMs (*n* = 3). Results were shown as mean ± SD. ^ns^*p* ≥ 0.05. Unpaired t-tests were used for the comparison of two groups.


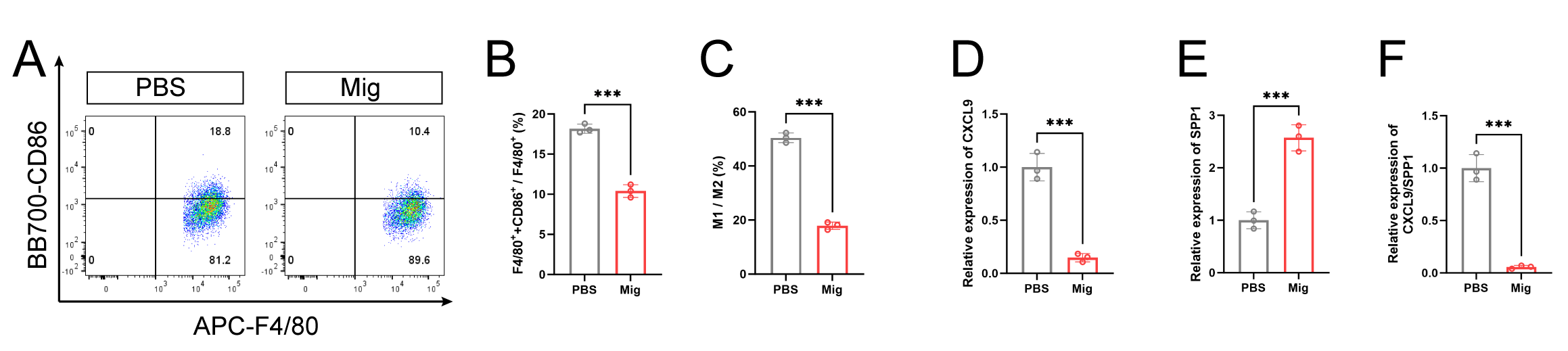


**Figure S7.** **Effect of OCDMs on CXCL9:SPP1 macrophage polarity and macrophages M1 polarization *in vitro*.**

(A-F) BMDMs treated with PBS or 10μg/ml migrasomes for 24 hours, followed by co-incubation with apoptotic OS cells for 24 hours. (A-C) Flow cytometry analysis of the proportion of M1-type macrophages and quantitative analysis (*n* = 3). (D-F) RT-qPCR analysis of the expression of CXCL9, SPP1 and CXCL9:SPP1 in BMDMs (*n* = 3). Results were shown as mean ± SD. ****p* < 0.001. Unpaired t-tests were used for the comparison of two groups.


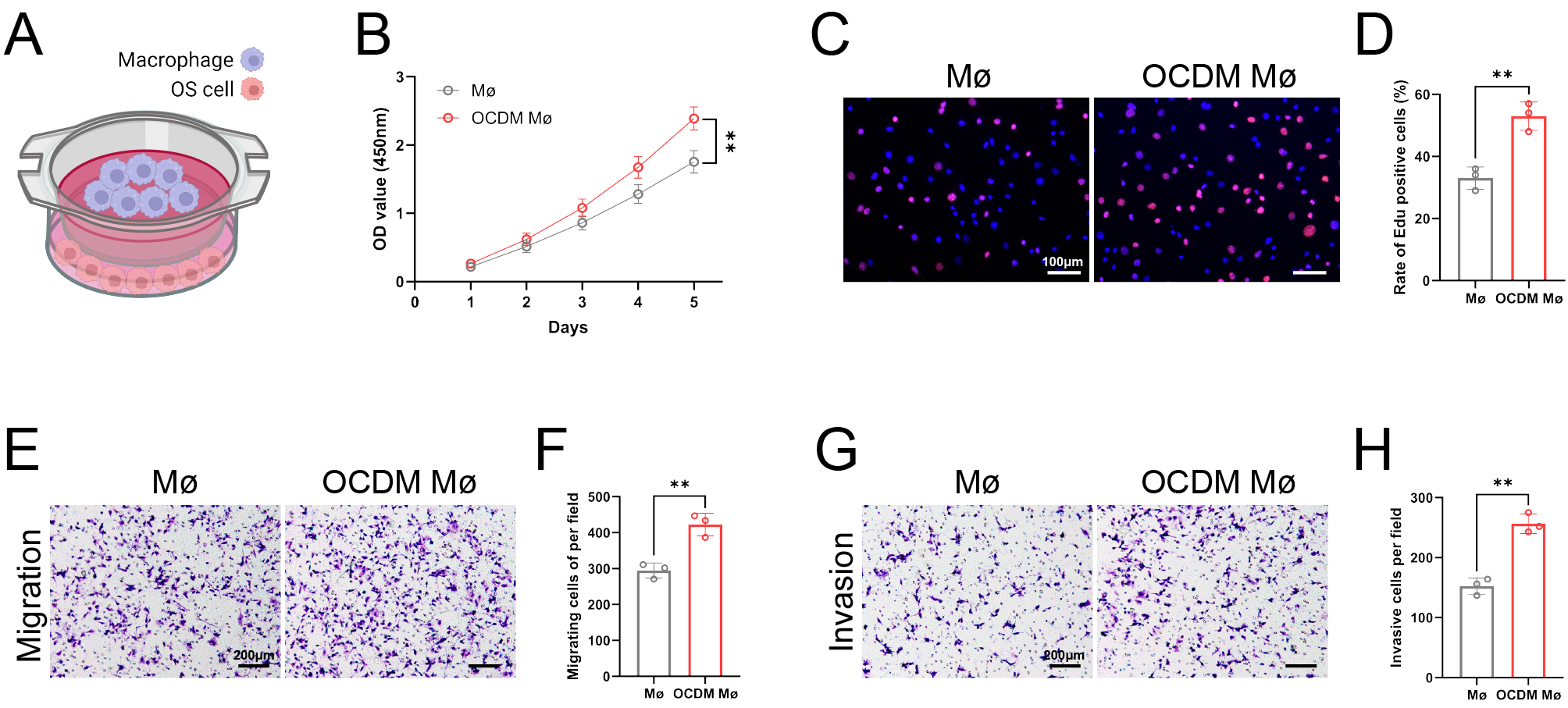


**Figure S8.** **OCDMs-treated macrophages promote OS cells proliferation, migration and invasion *in vitro*.**

(A-H) BMDMs treated with PBS or 10μg/ml migrasomes for 24 h were directly co-incubated with apoptotic OS cells for 24 h. Subsequently, BMDMs were co-incubated with OS cells for 48 h via co-culture chambers. (A) schematic of cell co-culture chamber (created with BioRender.com). (B) CCK8 analysis of OS cells (*n* = 3). (C, D) Representative images and quantitative analysis of OS cells EDU assays (*n* = 3). Scale bars = 100 µm. (E, F) Representative images and quantitative analysis of OS cells transwell migration assays (*n* = 3). Scale bars = 200 µm. (G, H) Representative images and quantitative analysis of OS cells transwell invasion assays (*n* = 3). Scale bars = 200 µm. Results were shown as mean ± SD. ***p* < 0.01. One-way ANOVA test or two-way ANOVA test was used for multivariate analysis. Unpaired t-tests were used for the comparison of two groups.


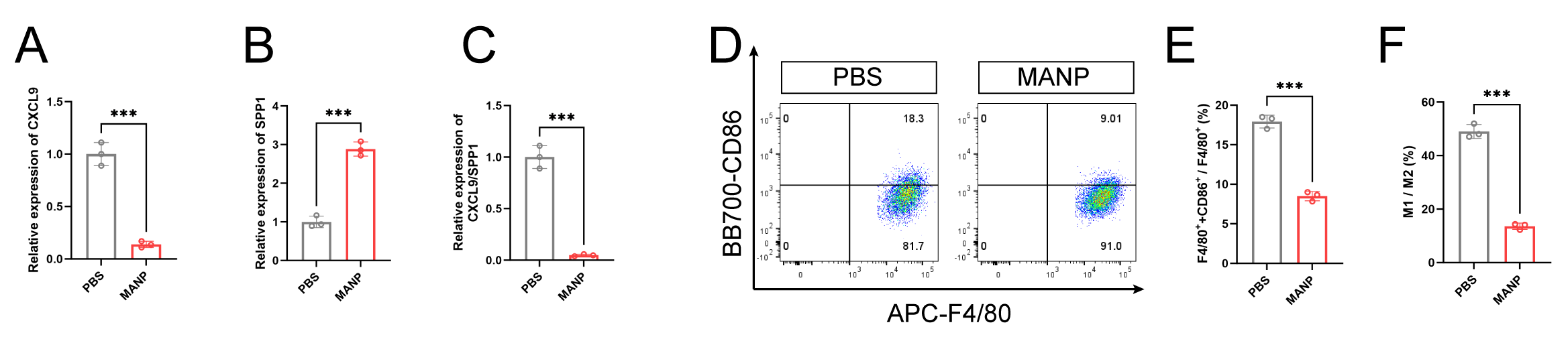


**Figure S9.** **Effect of MANPs on CXCL9:SPP1 macrophage polarity and macrophages M1 polarization *in vitro*.**

(A-F) BMDMs treated with PBS or 10μg/ml MANPs for 24 hours, followed by co-incubation with apoptotic OS cells for 24 hours. (A-C) RT-qPCR analysis of the expression of CXCL9, SPP1 and CXCL9:SPP1 in BMDMs (*n* = 3). (D-F) Flow cytometry analysis of the proportion of M1-type macrophages and quantitative analysis (*n* = 3). Results were shown as mean ± SD. ****p* < 0.001. Unpaired t-tests were used for the comparison of two groups.


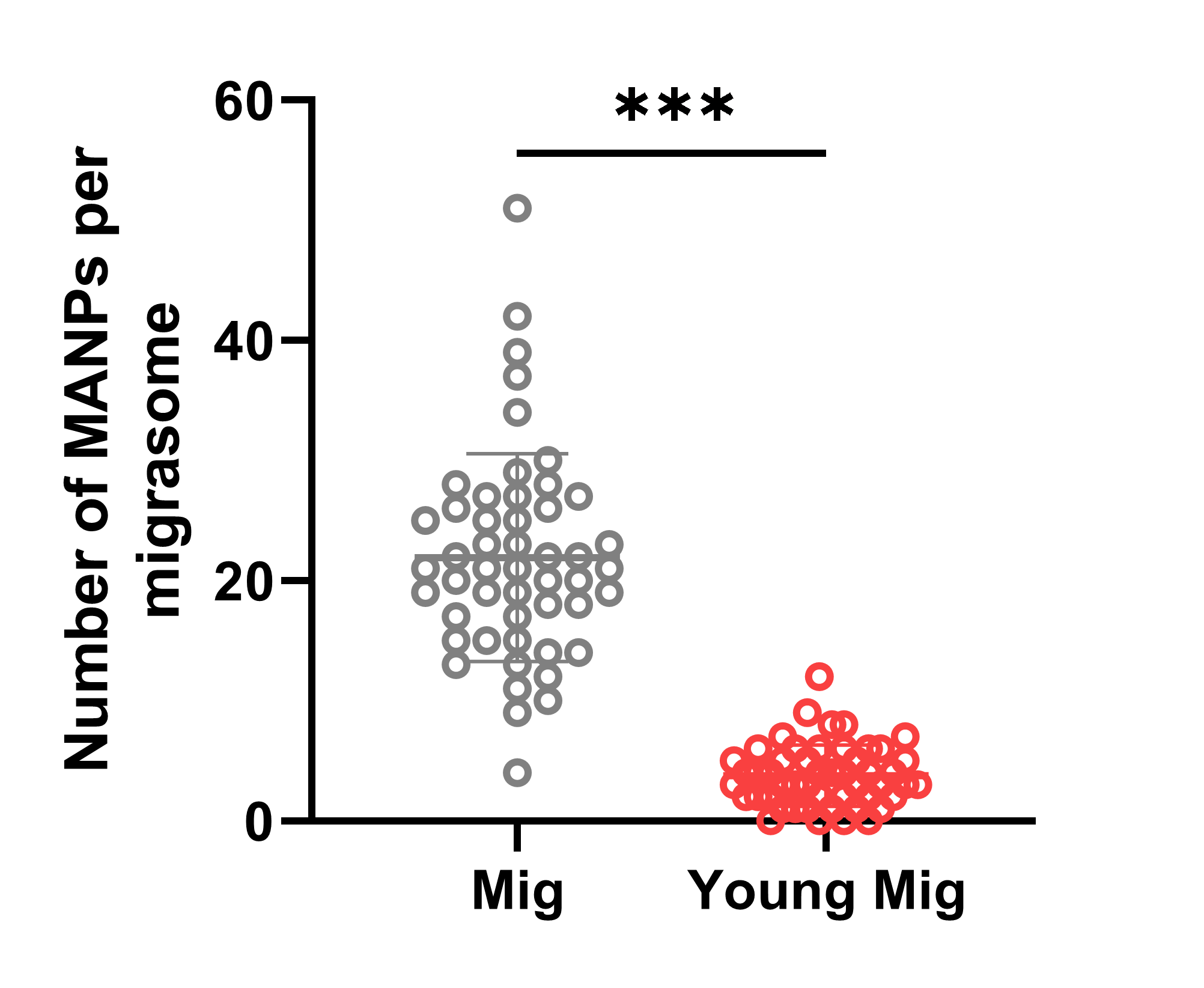


**Figure S10.** **Young migrasomes lack MANPs compared to migrasomes.**

Quantitative analysis of MANPs per migrasome (*n* = 50). Results were shown as mean ± SD. ****p* < 0.001. Unpaired t-tests were used for the comparison of two groups.


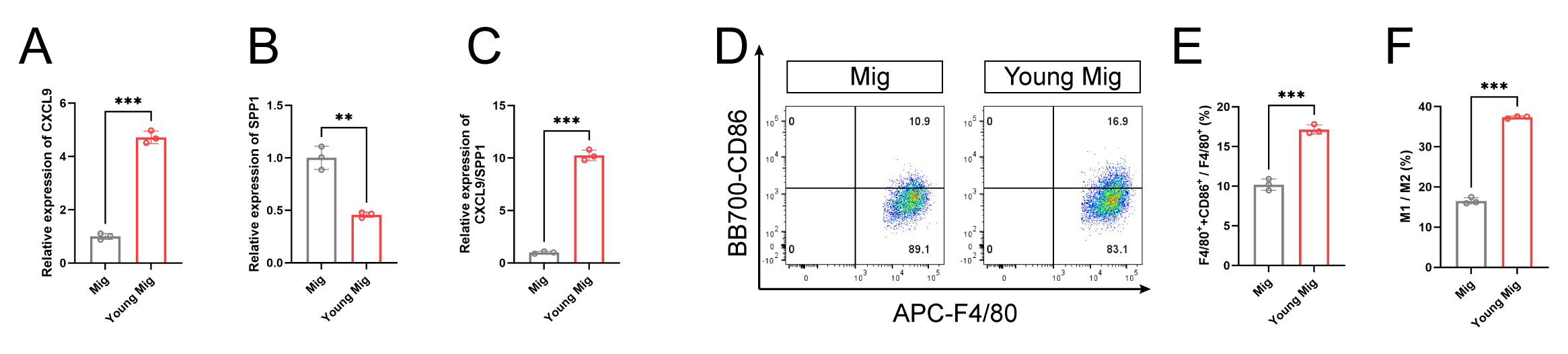


**Figure S11.** **Effect of young migrasomes on CXCL9:SPP1 macrophage polarity and macrophages M1 polarization *in vitro*.**

(A-F) BMDMs treated with 10μg/ml migrasomes or 10μg/ml young migrasomes for 24 hours, followed by co-incubation with apoptotic OS cells for 24 hours. (A-C) RT-qPCR analysis of the expression of CXCL9, SPP1 and CXCL9:SPP1 in BMDMs (*n* = 3). (D-F) Flow cytometry analysis of the proportion of M1-type macrophages and quantitative analysis (*n* = 3). Results were shown as mean ± SD. ***p* < 0.01, ****p* < 0.001. Unpaired t-tests were used for the comparison of two groups.


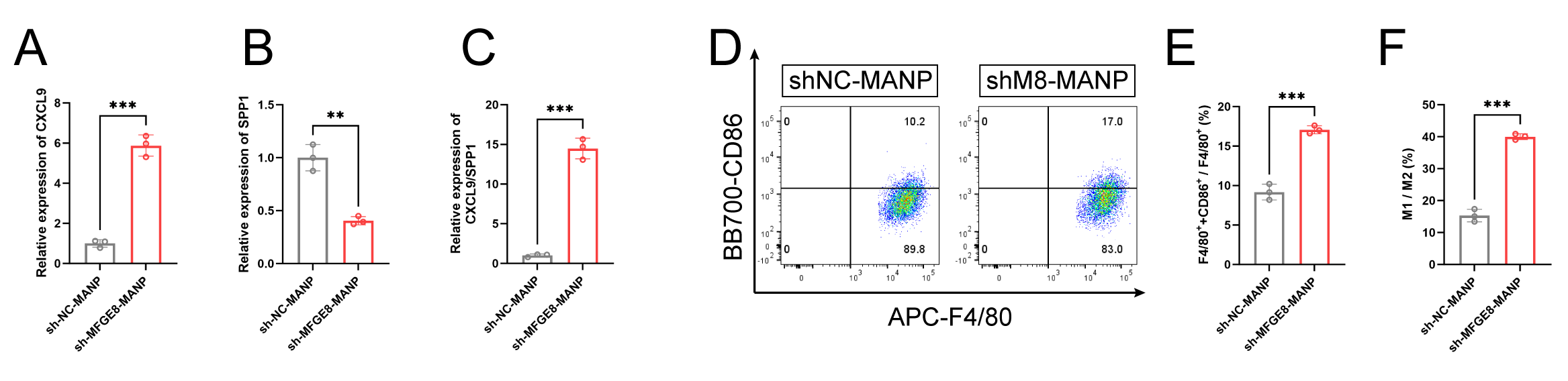


**Figure S12.** **Effect of sh-MFGE8-MANPs on CXCL9:SPP1 macrophage polarity and macrophages M1 polarization *in vitro*.**

(A-F) BMDMs treated with 10μg/ml shMFGE8-MANPs or 10μg/ml shNC-MANPs for 24 hours, followed by co-incubation with apoptotic OS cells for 24 hours. (A-C) RT-qPCR analysis of the expression of CXCL9, SPP1 and CXCL9:SPP1 in BMDMs (*n* = 3). (D-F) Flow cytometry analysis of the proportion of M1-type macrophages and quantitative analysis (*n* = 3). Results were shown as mean ± SD. ***p* < 0.01, ****p* < 0.001. Unpaired t-tests were used for the comparison of two groups.


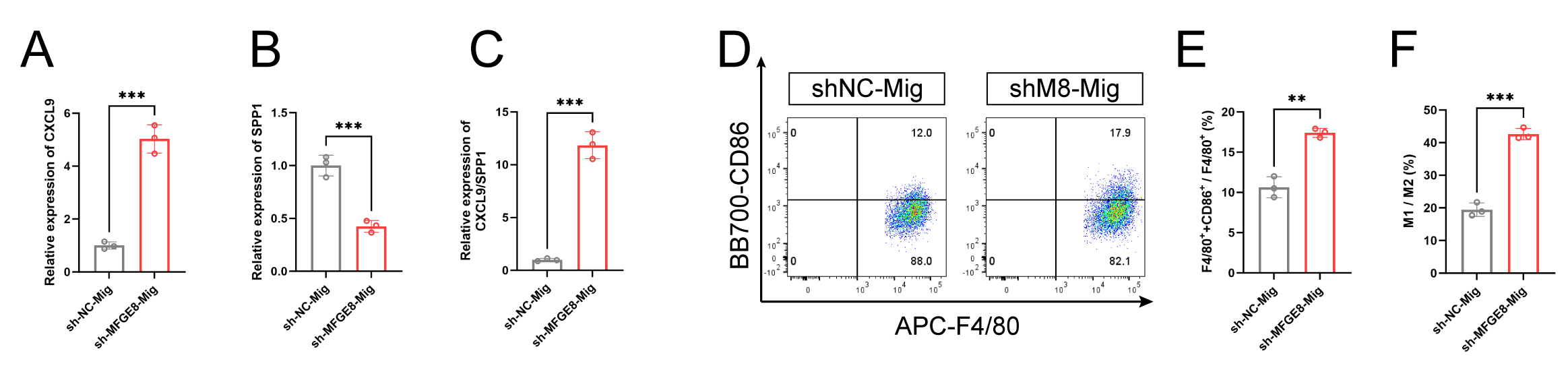


**Figure S13.** **Effect of sh-MFGE8-OCDMs on CXCL9:SPP1 macrophage polarity and macrophages M1 polarization *in vitro*.**

(A-F) BMDMs treated with 10μg/ml shMFGE8-migrasomes or 10μg/ml shNC-migrasomes for 24 hours, followed by co-incubation with apoptotic OS cells for 24 hours. (A-C) RT-qPCR analysis of the expression of CXCL9, SPP1 and CXCL9:SPP1 in BMDMs (*n* = 3). (D-F) Flow cytometry analysis of the proportion of M1-type macrophages and quantitative analysis (*n* = 3). Results were shown as mean ± SD. ***p* < 0.01, ****p* < 0.001. Unpaired t-tests were used for the comparison of two groups.


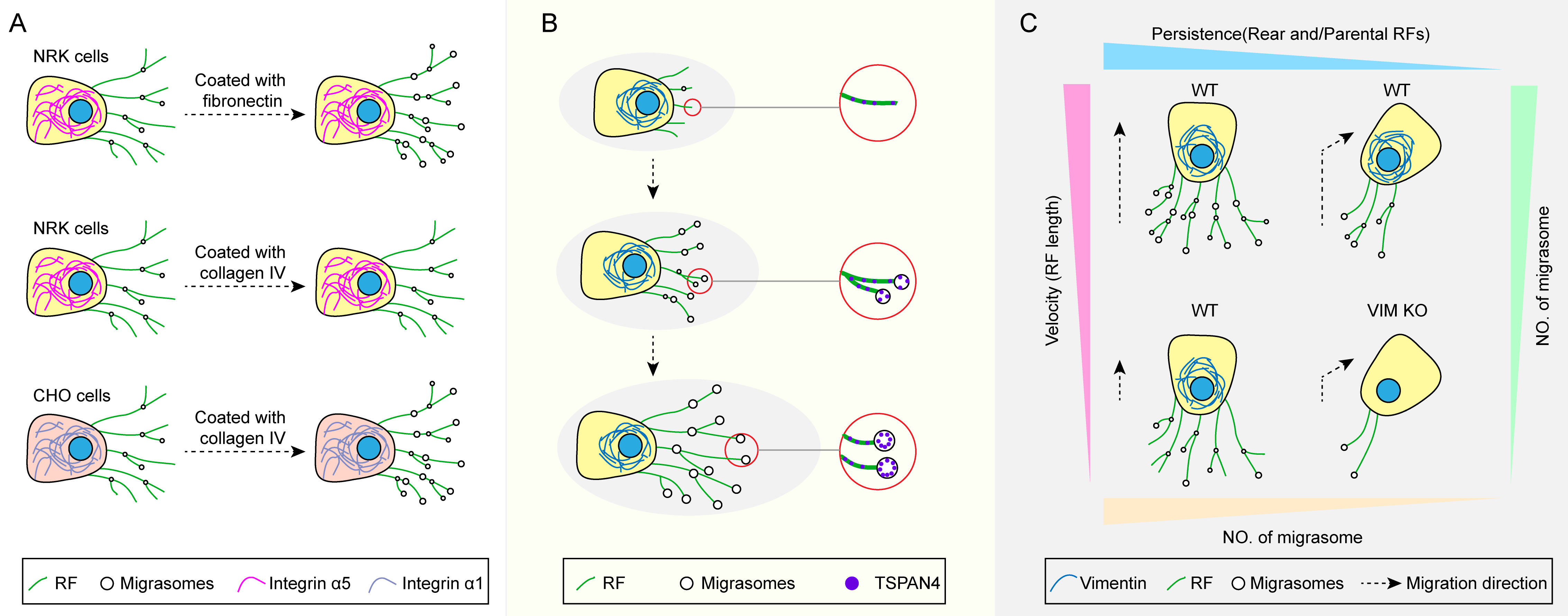


**Figure S14.** **Schematic illustration of the mechanisms of migrasome formation.**

(A) Schematic model of pairing of integrins with matched ECM ligand determines migrasome formation. (B) Schematic model of recruitment of TSPAN4 drives migrasome formation. (C) Schematic model of cell migration pattern to orchestrate migrasome formation.


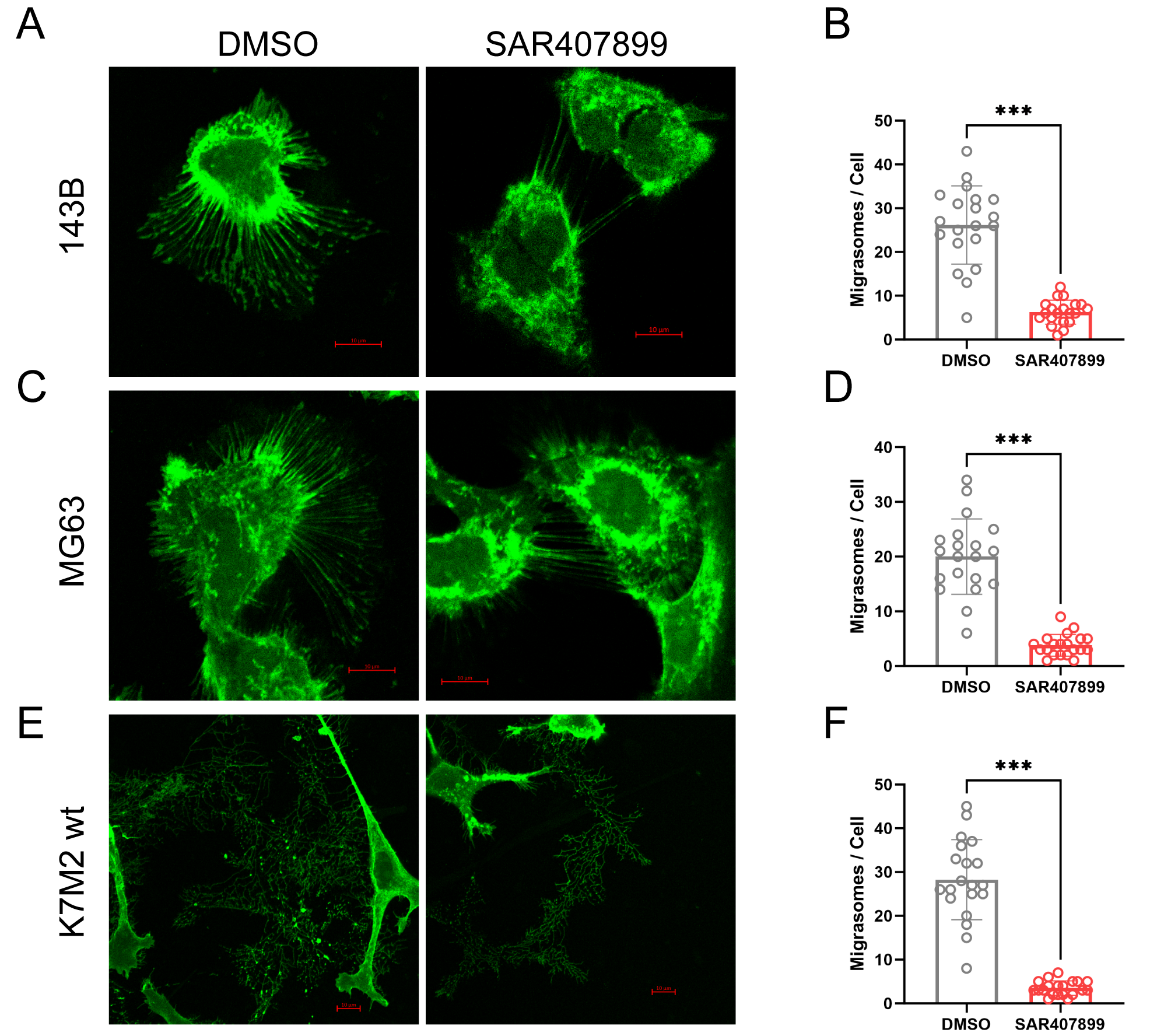


**Figure S15.** **SAR407899 inhibits the formation of migrasomes in OS cells.**

(A-F) Representative confocal images of OS cells treated with DMSO or 10 µM SAR407899 and quantification of the average migrasome number per cell (*n* = 20). Scale bars = 10 µm. Results were shown as mean ± SD. ****p* < 0.001. Unpaired t-tests were used for the comparison of two groups.


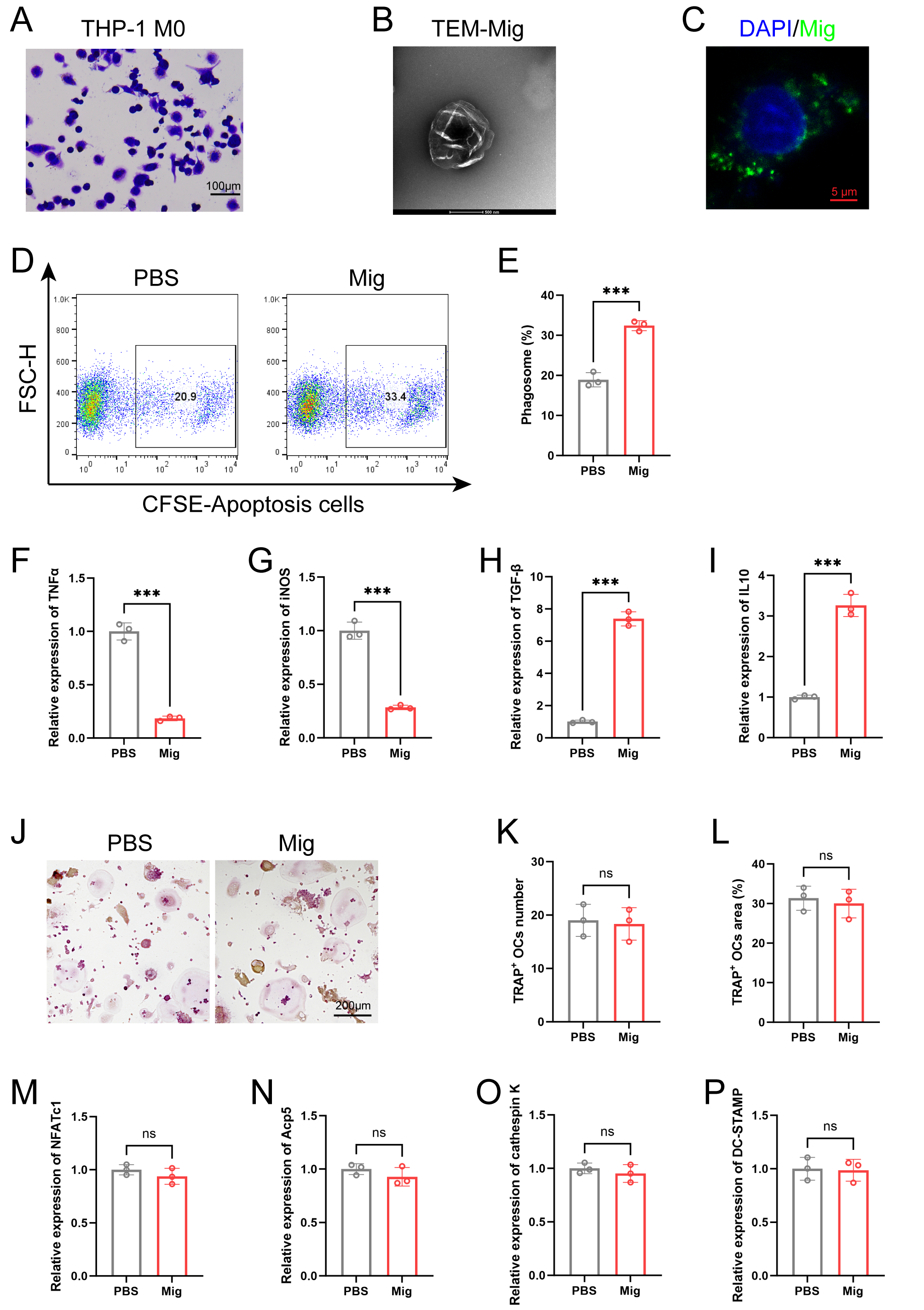


**Figure S16.** **Effect of migrasomes on THP-1-derived macrophages and osteoclasts.**

(A) The morphology of M0 (non-polarized THP-1 cells). Scale bars = 100 µm. (B) Representative TEM images of purified migrasomes from 143B cells by negative staining (*n* = 3). Scale bars = 500 nm. (C) Representative immunostaining images of macrophages phagocytosis of migrasomes (*n* = 3). Scale bars = 5 µm. (D-E) After treated with PBS or 10μg/ml migrasomes for 24 hours, flow cytometry analysis of the proportion of macrophages that phagocytosis of apoptotic OS cells and quantitative analysis (*n* = 3). (F-I) After treated with PBS or 10μg/ml migrasomes for 24 hours, followed by co-incubation with apoptotic OS cells for 24 hours. RT-qPCR analysis of the expression of M1 and M2 polarization markers in macrophages (*n* = 3). (J) Representative TRAP staining images of osteoclasts administered with PBS or 10μg/ml migrasomes (*n* = 3). Scale bars = 200 µm. (K-L) Number and area of multi-nucleated TRAP^+^ cells with indicated treatment (*n* = 3). (M-P) RT-qPCR analysis of the expression of osteoclastspecific genes NFATc1, Acp5, cathespin K and DC-STAMP in osteoclasts administered with PBS or 10μg/ml migrasomes (*n* = 3). Results were shown as mean ± SD. ^ns^*p* ≥ 0.05, ****p* < 0.001. Unpaired t-tests were used for the comparison of two groups.


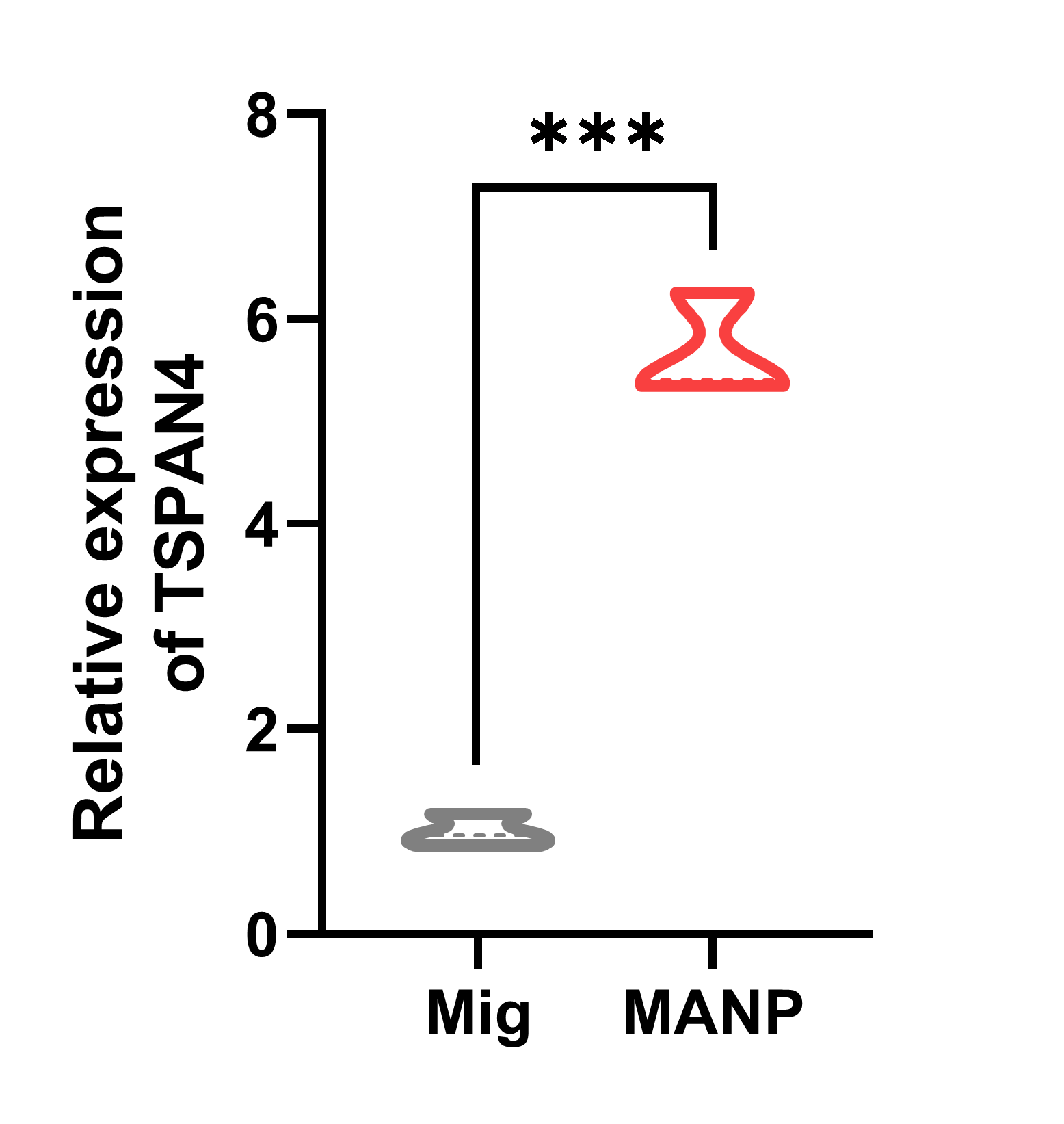


**Figure S17.** **TSPAN4 expression levels are significantly higher in MANPs than in migrasomes.**

Relative expression of TSPAN4 from 4D proteome sequencing (*n* = 3). Results were shown as mean ± SD. ****p* < 0.001. Unpaired t-tests were used for the comparison of two groups.


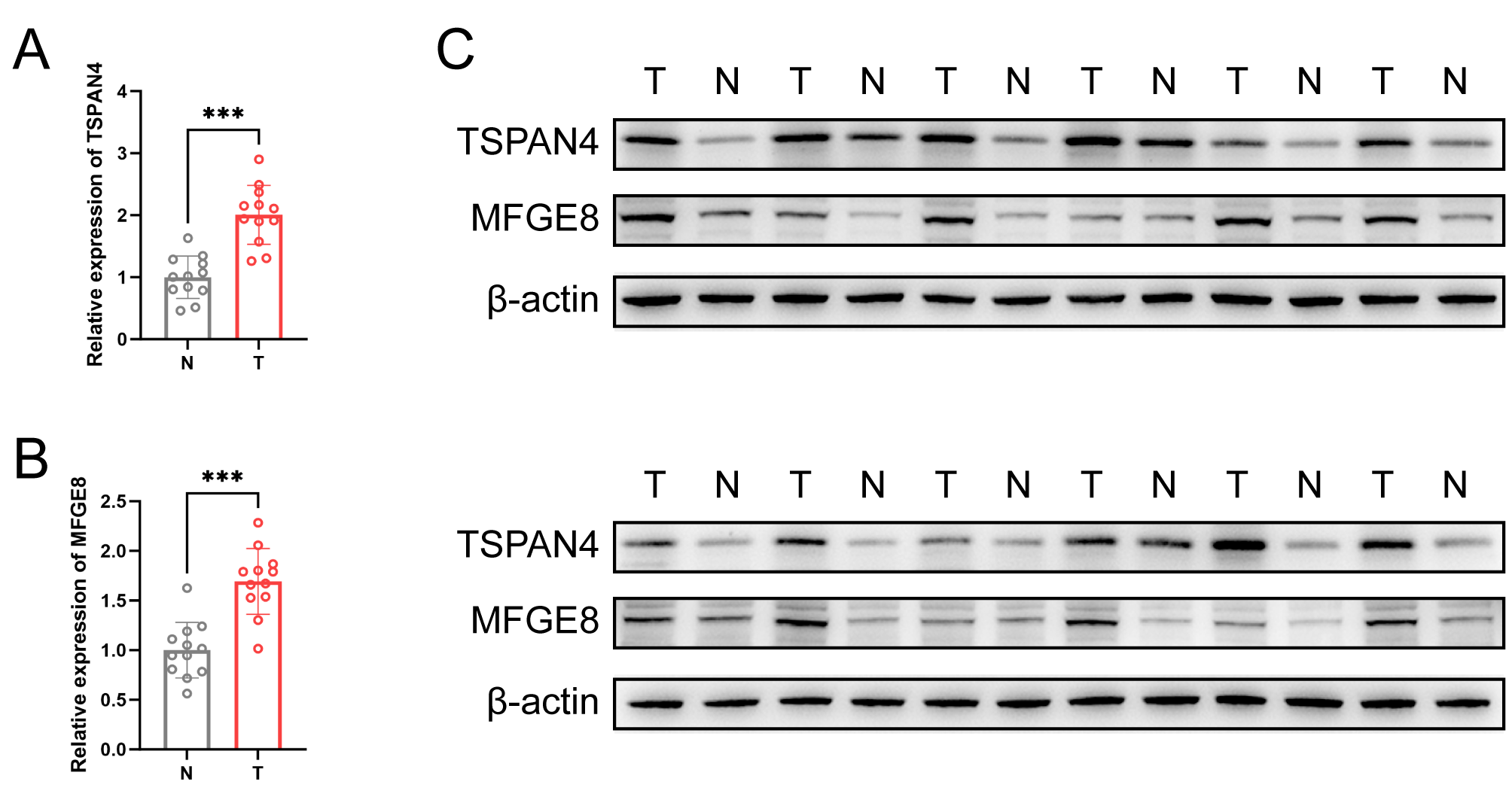


**Figure S18.** **TSPAN4 and MFGE8 are highly expressed in osteosarcoma.**

(A, B) The expression of TSPAN4 and MFGE8 were examined in twelve pairs of OS tissues and matched non-tumor tissues by RT-qPCR (*n* = 12). (C) The expression of TSPAN4 and MFGE8 were determined in twelve pairs of OS tissues and matched non-tumor tissues by Western blot (*n* = 12). Results were shown as mean ± SD. ****p* < 0.001. Unpaired t-tests were used for the comparison of two groups.


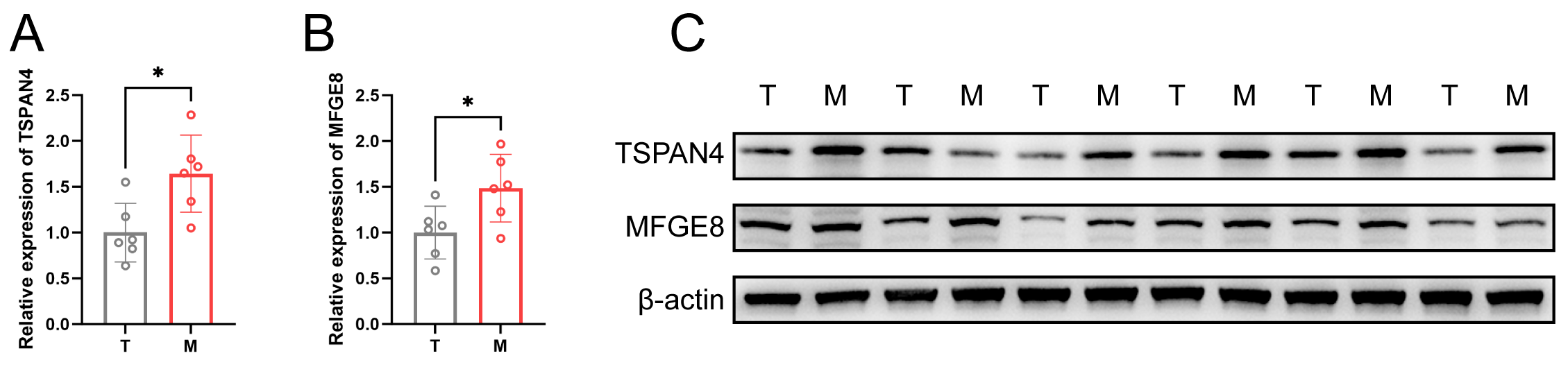


**Figure S19.** **TSPAN4 and MFGE8 are highly expressed in metastatic osteosarcoma.**

(A, B) The expression of TSPAN4 and MFGE8 were examined in six pairs of OS tissues and matched lung metastatic tissues by RT-qPCR (*n* = 6). (C) The expression of TSPAN4 and MFGE8 were determined in six pairs of OS tissues and matched lung metastatic tissues by Western blot (*n* = 6). Results were shown as mean ± SD. **p* < 0.05. Unpaired t-tests were used for the comparison of two groups.


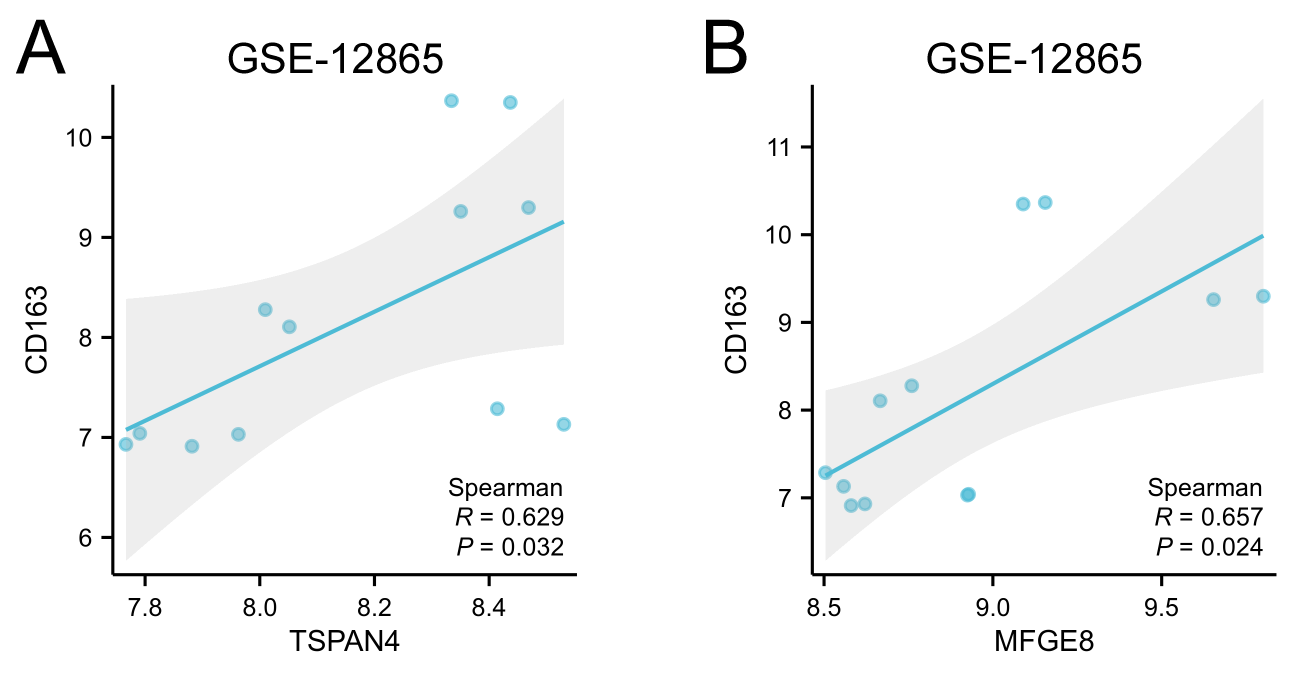


**Figure S20.** **Correlation between TSPAN4/MFGE8 and CD163 in OS samples from GSE12865.**

(A) Correlation between the expression of TSPAN4 and CD163 in OS samples from GSE12865. (B) Correlation between the expression of MFGE8 and CD163 in OS samples from GSE12865. Correlation analysis was conducted by the Spearman method.

Table S1. Primers used for qPCR.

| Target gene | Species | Forward | Reverse |
| --- | --- | --- | --- |
| *TSPAN4*  *TNFα* | Mouse  Mouse | ATGTTCGCCTTCAACCTGCTCTTC  ACGCTCTTCTGTCTACTGAACTTCG | GTTGGCAGCCGACAAGGATGG  TGGTTTGTGAGTGTGAGGGTCTG |
| *INOS* | Mouse | GACGAGACGGATAGGCAGAGATTG | AACTCTTCAAGCACCTCCAGGAAC |
| *CD86*  *CD206*  *CD163*  *IL-4* | Mouse  Mouse  Mouse  Mouse | GAGCACTATTTGGGCACAGAGAAAC  TGTACGCAGTGGTTGGCAGTG  CCTCCTCATTGTCTTCCTCCTGTG  GTTGTCATCCTGCTCTTCTTTCTCG | TGAAGTCGTAGAGTCCAGTTGTTCC  GCTCTGATGATGGACTTCCTGGTAG  CATCCGCCTTTGAATCCATCTCTTG  CATGGCGTCCCTTCTCCTGTG |
| *MFGE8* | Mouse | TCCGCCTCGTCTGTGTATATGG | TTGCTATCATAGTTGCTGGCTGTC |
| *NFATc1* | Mouse | GGAGAGTCCGAGAATCGAGAT | TTGCAGCTAGGAAGTACGTCT |
| *Acp5* | Mouse | AGCAGCTCCCTAGAAGATGGA | AGCCACAAATCTCAGGGTGG |
| *CathespinK* | Mouse | GGAGTTGACTTCCGCAATCCT | ACTTGAACACCCACATCCTGC |
| *DC-STAMP* | Mouse | CCGTGAAGGTAGGAACGCTT | AGATTCAGCGGAGTGGCAAG |
| *SPP1* | Mouse | AAGAGCGGTGAGTCTAAGGAGTCC | TGGCTGCCCTTTCCGTTGTTG |
| *CXCL9* | Mouse | AGCCGAGGCACGATCCACTAC | AGGCAGGTTTGATCTCCGTTCTTC |
| *β-actin* | Mouse | GATGGTGGGAATGGGTCAGAAGG | TTGTAGAAGGTGTGGTGCCAGATC |
| *TSPAN4* | Human | GCCTACACGGACAAGATTGACAG | AGCGGAAGTCGGTCTGGATG |
| *MFGE8* | Human | ATCGCCGCCTCGTCTGTG | CGTCATTGCTGCTGGGTGTC |
| *TNFα* | Human | TGGCGTGGAGCTGAGAGATAACC | CGATGCGGCTGATGGTGTGG |
| *INOS* | Human | CAGGGTGGAAGCGGTAACAAAGG | CCTGCTTGGTGGCGAAGATGAG |
| *TGF-β* | Human | AGCAACAATTCCTGGCGATACCTC | TCAACCACTGCCGCACAACTC |
| *IL-10* | Human | GCCAAGCCTTGTCTGAGATGATCC | GCCTTGATGTCTGGGTCTTGGTTC |
| *β-actin* | Human | GGCCAACCGCGAGAAGATGAC | GGATAGCACAGCCTGGATAGCAAC |
